# HIV-1 p24Gag adaptation to modern and archaic HLA-allele frequency differences in ethnic groups contributes to viral subtype diversification

**DOI:** 10.1093/ve/veaa085

**Published:** 2020-12-12

**Authors:** Nicolaas C Kist, Ben Lambert, Samuel Campbell, Aris Katzourakis, Daniel Lunn, Philippe Lemey, Astrid K N Iversen

**Affiliations:** Division of Clinical Neurology, Nuffield Department of Clinical Neurosciences, Weatherall Institute of Molecular Medicine, University of Oxford, Oxford OX3 9DS, UK; Department of Zoology, University of Oxford, South Parks Road, Oxford OX1 3PS, UK; Department of Zoology, University of Oxford, South Parks Road, Oxford OX1 3PS, UK; Department of Infectious Disease Epidemiology, School of Public Health, Faculty of Medicine, Imperial College London, Medical School Building St Mary’s Campus, Norfolk Place, London W2 1PG, UK; Division of Clinical Neurology, Nuffield Department of Clinical Neurosciences, Weatherall Institute of Molecular Medicine, University of Oxford, Oxford OX3 9DS, UK; Department of Zoology, University of Oxford, South Parks Road, Oxford OX1 3PS, UK; Department of Zoology, University of Oxford, South Parks Road, Oxford OX1 3PS, UK; Department of Statistics, University of Oxford, St Giles’, Oxford OX1 3LB, UK; Department of Microbiology and Immunology, Rega Institute for Medical Research, KU Leuven - University of Leuven, Leuven B-3000, Belgium; Division of Clinical Neurology, Nuffield Department of Clinical Neurosciences, Weatherall Institute of Molecular Medicine, University of Oxford, Oxford OX3 9DS, UK

**Keywords:** HIV-1, adaptation, subtype, diversification, HLA, phylodynamics, phylogenetics

## Abstract

Pathogen-driven selection and past interbreeding with archaic human lineages have resulted in differences in human leukocyte antigen (HLA)-allele frequencies between modern human populations. Whether or not this variation affects pathogen subtype diversification is unknown. Here we show a strong positive correlation between ethnic diversity in African countries and both human immunodeficiency virus (HIV)-1 p24*gag* and subtype diversity. We demonstrate that ethnic HLA-allele differences between populations have influenced HIV-1 subtype diversification as the virus adapted to escape common antiviral immune responses. The evolution of HIV Subtype B (HIV-B), which does not appear to be indigenous to Africa, is strongly affected by immune responses associated with Eurasian HLA variants acquired through adaptive introgression from Neanderthals and Denisovans. Furthermore, we show that the increasing and disproportionate number of HIV-infections among African Americans in the USA drive HIV-B evolution towards an Africa-centric HIV-1 state. Similar adaptation of other pathogens to HLA variants common in affected populations is likely.

## 1. Introduction

Differences in human leukocyte antigen (HLA) frequencies between geographically dispersed populations are primarily the result of pathogen-driven selection (Doherty and Zinkernagel 1975) and past interbreeding with archaic human lineages (Segurel and Quintana-Murci 2014; Quintana-Murci 2019). Although some gene-flow from modern human into Neanderthals appears to have occurred in Europe as early as 100,000–200,000 years before present (YBP) (Chen et al. 2020), the major dispersal of modern humans from Africa into Eurasia took place ∼60,000 YBP (Reich 2018). These modern humans first mated with Neanderthals in the Levant and thousands of years later with at least three distinct Denisovan lineages in Western Asia, East Asia, and Oceania (Reich 2018; Jacobs et al. 2019). Because these groups of archaic humans had already adapted to Eurasian pathogens for ∼700,000–450,000 years, the introgression (the gaining of genetic material through interbreeding) of archaic HLA variants likely provided modern humans with pre-adapted HLA variants that were crucial to their survival in the new environments. This survival advantage is evident today as HLA Class I alleles of archaic origin comprise at least 50–85 per cent of the HLA variants in Eurasian populations, even though the overall archaic genomic inheritance is only 1–6 per cent (Abi-Rached et al. 2011). Despite some back-migration of Eurasians to Africa over the last ∼20,000 years (Chen et al. 2020), these archaic HLA variants are the key difference between the HLA profile of populations in Africa and Eurasia. Statistical analyses of African whole-genome sequencing data provide evidence of interbreeding with other lineages of archaic humans within Africa, but the results are controversial because of a lack of fossil DNA (Hsieh et al. 2016; Nielsen et al. 2017). What impact the HLA variation between populations over time has had on the subtype diversification of both old and novel pathogens such as human immunodeficiency virus (HIV)-1 is largely unknown.

The cross-species transmission of simian immunodeficiency virus that gave rise to the HIV-1 group responsible for the majority of the global HIV-1 epidemic (Group M), likely occurred ∼1900 in Cameroon or the Democratic Republic of the Congo (DRC; Worobey et al. 2008). However, the HIV-1 pandemic only ignited in Kinshasa in the DRC in the 1920s and spread over the following decades via transport networks and migrant labour throughout the country and beyond (Faria et al. 2014). HIV-1 started to diversify into subtypes around 1960 concurrent with the transition from a slow to a much faster transmission phase (Faria et al. 2014).

HIV-1 subtypes are defined using phylogenetic analyses and the genetic distance between them is comparable; they are labelled alphabetically, and each subtype consensus sequence carries distinct combinations of subtype-specific amino acids in conserved subtype-specific positions in every viral protein in addition to changes at other positions (Foley et al. 2018). These subtype-specific amino acid differences are particularly striking in the conserved, clinically important, highly immunogenic cytotoxic T lymphocyte (CTL) target regions of p24Gag (the capsid protein) where they constitute the only difference between the subtype consensus sequences (Supplementary Fig. S1; Kiepiela et al. 2007; Matthews et al. 2008; Goulder and Walker 2012).

The highest number of HIV-1 subtypes and circulating and unique recombinant forms (CRFs and URFs) of various HIV subtypes is found within Africa (Chang et al. 2015; Foley et al. 2018). Whereas subtype-precursor lineages to a high degree are embedded within the larger HIV-1 diversity in the DRC, which also includes examples of most global subtypes, only distinct subtypes predominate outside the country (Rambaut et al. 2001; Faria et al. 2014). The most geographically widespread subtype is HIV-B (Hemelaar et al. 2011). The ancestral HIV-B virus originated in Africa and spread to Haiti around 1965–70 (Gilbert et al. 2007). The earliest HIV-B sequences in the USA were detected in stored serum samples collected from men-who-have-sex-with-men (MSM) in 1978–79 (Gilbert et al. 2007; Worobey et al. 2016). HIV-B spread within the USA and beyond and now accounts for ∼11 per cent of worldwide infections, although no indigenous (i.e. not imported) HIV-B subtype has ever been identified in Africa (Hemelaar et al. 2011). In contrast, an HIV-C-precursor lineage spread successfully from the southern DRC Katanga province to most of the Southern and Eastern parts of Africa, and today HIV-C is responsible for ∼50 per cent of all infections (Hemelaar et al. 2011; Faria et al. 2014).

Upon infection, HLA Class I (A–C) molecules on the cell surface present peptide fragments (epitopes) from viral proteins that have been digested by intra-cellular proteasomes. This presentation is a central point in the interplay between HIV-1 and the host’s immune system as it triggers CTL and Natural Killer cell responses that can kill HIV-infected cells. The HLA genes are highly polymorphic, and an HLA molecule has specific binding motifs that allow only some of the produced epitopes to be presented by each HLA variant. Because the combination of HLA variants (or the HLA profile) varies between individuals, the selective pressures on the virus vary accordingly (Tenzer et al. 2014). The variation in HLA frequencies between populations and ethnic groups ensures that the sum of the HLA-associated selective pressures on the virus will also differ.

HLA-associated polymorphisms have primarily been examined within distinct HIV-1 subtypes in combined Canadian/US HIV-B sequence sets (Cotton et al. 2014; Kinloch et al. 2016) after Bhattacharya et al. (2007) reported that viral subtype effects confounded previously reported HLA associations (Moore et al. 2002). Many of the polymorphisms are intra-epitope CTL escape mutations, and studies have focussed on the presence, accumulation, and possible reversion of small subsets of these mutations (Supplementary Fig. S2A; Leslie et al. 2004, 2005; Kawashima et al. 2009; Fryer et al. 2012) and their impact on viral fitness and viral load set point (Matthews et al. 2008; Carlson et al. 2015). Although HLA-associated polymorphisms are found in all HIV-1 proteins, analyses of the specificity and breadth of CTL responses associated with immune control of HIV-1 have demonstrated that responses targeting p24Gag were almost exclusively associated with lowering viremia (Kiepiela et al. 2007; Matthews et al. 2008; Goulder and Walker 2012).

We experimentally identified a second form of HLA-associated selection which occur at subtype-specific positions in p24Gag and affects proteasomal production of all epitopes encoded in epitope-clusters up- and down-stream of the subtype-specific position (illustrated in Supplementary Fig. S2B and explained in detail in the legend; Tenzer et al. 2014). These epitope-clusters typically contain ∼20–50 epitopes presented by approximately as many HLA variants and proteasomal production of each epitope is either increased, unaffected, decreased, or eliminated depending on the nature of the amino acid at the subtype-specific position. We found that intra-host selection on HIV-1 favoured an amino acid at the subtype-specific position which thwarted or limited production of HIV-1 epitopes that that individual’s HLA variants could present regardless of the infecting subtype. At the population level, we observed an inverse relationship between the amount of each epitope produced following processing of the HIV-B or -C consensus sequences and the frequencies of the presenting HLA variants in the populations in which these subtypes circulated (Supplementary Fig. S2C and D; Tenzer et al. 2014).

Intra-host selection on HIV-1 to escape anti-viral CTL responses through intra-epitope escape-mutations and changes at subtype-specific sites that limit epitope-processing likely co-occurs. However, within the conserved, highly immunogenic, p24Gag regions (Supplementary Fig. S1), subtype-specific positions are subjected to the combined pressure by >80 HLA variants (HIV Molecular Immunology Compendium 2016) and changes at these positions at the HIV-1 consensus level are likely observed faster than changes within single epitopes restricted by one, or a few, HLA variants (Kawashima et al. 2009).

The current model for HIV-1 subtype diversification is that HIV-1 subtypes are the results of multiple, selectively neutral, random ‘founder events’, whereby individuals with distinct viral lineages moved to new regions and established local epidemics (Pybus and Rambaut 2009; Peeters, Jung, and Ayouba 2013). HLA variation and hence variation in immune response targeting between populations are generally not assumed to play a significant role in this process (Pybus and Rambaut 2009; Peeters, Jung, and Ayouba 2013). Here we considered the effect of HLA variation between populations and HLA-associated selection at subtype-specific sites through modification of epitope production (Tenzer et al. 2014).

First, we investigated whether differences in ethnic diversity in African countries was associated with HIV-1 p24Gag diversity (on the amino-acid level ignoring subtype) and subtype diversity, respectively, and observed robust positive linear relationships. Second, we explored the HLA profile patterns in all HIV-B and HIV-C-infected patients with linked HLA information using an HLA-based principal component analysis (PCA). We found that the most influential drivers in principal component (PC)1 were Eurasian HLA variants introgressed from archaic humans, whereas HLA variants associated with the two major haplotypes in South Africa strongly influenced PC2. Third, we estimated which of the >80 epitope-presenting HLA variants in the clinically important HIV-B and HIV-C p24Gag regions had the most substantial effect on the evolution of the five subtype-specific sites within these regions. Fourth, we examined if the continuously increasing proportion of African Americans in the USA HIV-B infected population over time affected the evolution of these subtype-specific sites. We found that the subtype-specific sites in HIV-B-p24Gag in the USA over time increasingly incorporated amino acids more commonly found in African HIV-1 subtypes than in the HIV-B consensus sequence.

Collectively, our results suggest a modified model for HIV-1 subtype diversification. We propose that variation at subtype-specific sites in HIV-1 p24Gag results from a combination of global HIV-1 spread through random founder events and the subsequent continuous granular viral adaptation to the HLA profile of the infected population.

## 2. Results

### 2.1 African HIV-1 p24Gag and subtype diversity correlate with ethnic diversity

We hypothesized that HLA-mediated selection drove HIV-1 p24Gag diversification and that subtype-specific amino acid differences were the result of selection for HIV-1 sequences that limited or abrogated processing of the epitopes presented by the most common HLA variants in each population. If HLA-mediated selection drove HIV-1 diversification, we should find greater HIV-1 diversity in countries with greater HLA diversity.

To investigate, we examined HIV-1 diversity within Africa where modern humans originated and have maintained relatively large effective population sizes for ∼300,000 years resulting in a high level of genetic diversity within and between the ∼2000 distinct African ethnic groups (Felix and Meur 2001; Patin et al. 2017; Tishkoff et al. 2009; Hershkovitz et al. 2018). Unfortunately, HLA data from Africa is limited as gold standard HLA A, B, and C data (i.e. all HLA types being reported as four-digit HLA variants) from only eight Sub-Saharan African populations/ethnic groups are available in the HLA-allele database (Gonzalez-Galarza et al. 2015; Supplementary Table S1). As a proxy for HLA diversity we used the Shannon entropy of each country’s ethnic demographics reported by Alesina et al. (2003) and Busby et al. (2017), which is based on ethnic, linguistic, cultural, and religious fractionalization.

Using this measure of ethnic diversity as a proxy for HLA diversity is justified by multiple studies that have demonstrated that African population structure largely mirrors linguistic and geographic similarity (Tishkoff et al. 2009; Busby et al. 2016, 2017) and have provided evidence of a strong link between genetic diversity and ethno-linguistic diversity (Busby et al. 2016, 2017; Excoffier et al. 1987, 1991; Tishkoff et al. 2009; Pagani et al. 2012; Patin et al. 2017). This genetic diversity is even higher in the HLA region as local adaptation at HLA loci generally are more pronounced than in the rest of the genome due to strong selective pressures by local pathogens and adaptive admixture between expanding populations and adapted local groups (Cao et al. 2004; Busby et al. 2017; Patin et al. 2017; Meyer et al. 2018). The strong link between ethnicity, genetics, and linguistics is not found on all continents as, for example, in South America, ethnic groups are largely defined by racial or physical criteria, not linguistics (Alesina et al. 2003).

**Figure 1.**
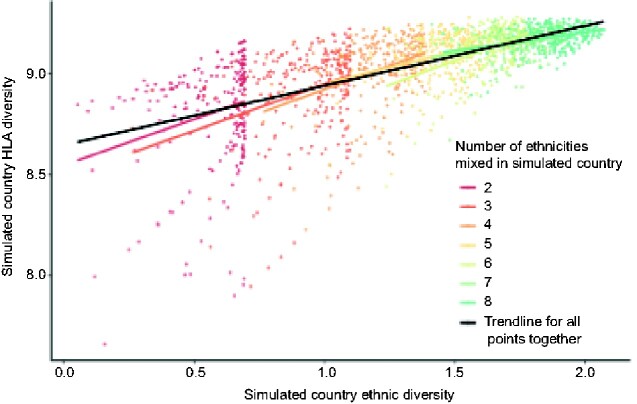
The simulated ethnic diversity of a fictitious country correlates with its simulated HLA diversity. Eight ‘populations’ were mixed in arbitrary proportions to simulate the mixture of ethnic groups within different fictive African ‘countries’. We then mixed the real gold standard HLA-allele frequency data from the eight populations in the HLA-allele database (Gonzalez-Galarza et al. 2015; Supplementary Table S1) according to the simulated proportions and performed linear regressions of the HLA diversity of these ‘simulated countries’ versus the simulated countries’ ethnic diversity calculated from the simulated proportions. Every point represents a simulated country; 500 countries were simulated for each number (k) of ethnic groups between 2 and 8 within each country. Each country consists of k randomly chosen populations/ethnic groups from Supplementary Table S1. Black line: best-fit line following a linear regression analysis of all simulated countries (*P* < 2 × 10^−16^). Coloured lines: best-fit line following a separate linear regression analysis of simulated countries consisting of k ‘ethnic groups’.

To further justify using ethnic diversity of African countries as a proxy for HLA diversity, we performed simulations showing a linear relationship between different mixtures of fictive ethnic groups and HLA diversity using the HLA A, B, and C data from the eight Sub-Saharan African populations/ethnic groups (Fig. 1).

Pooling our results across all replicates and numbers of ethnic mixing populations, we found a strong and statistically significant relationship between ethnic diversity and HLA diversity (*R*^2^ = 0.41, *F*_1, 1,748_ = 1,190, *P* < 2 × 10^−16^). Furthermore, this result held for subsets of the data comprising simulations of populations from a given number of ethnicities. Collectively, these simulations underscored that ethnic diversity in Africa is a valid proxy for HLA diversity.

**Figure 2.**
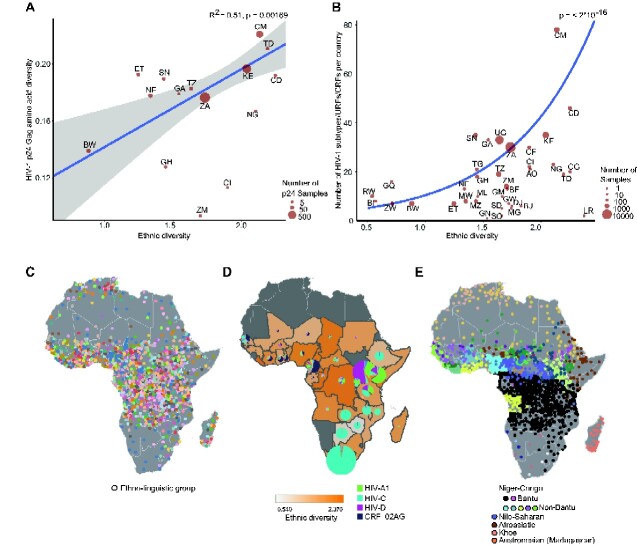
Ethnic diversity drives HIV-1 p24Gag and subtype diversification. (A) Weighted linear regression of HIV-1 p24Gag diversity versus ethnic diversity (Alesina et al. 2003; each determined using Shannon entropy). All Sub-Saharan African countries with ≥ 6 p24Gag sequences were included (Foley et al. 2018) (the number of sequences and the ISO alpha-2 two-letter country codes are shown in Supplementary Table S2**).** Grey-shaded area represents the 95 per cent confidence interval (CI). (B). Weighted linear regression of the count of HIV-1 subtypes, URFs and CRFs (Foley et al. 2018) versus ethnic diversity (Alesina et al. 2003) within Sub-Saharan Africa (illustrated in D) using a generalized linear model fit with a Poisson error distribution with a log link function (the number of subtypes, CRFs, and URFs and the ISO alpha-2 two-letter country codes are shown in Supplementary Table S2). Grey-shaded area represents the 95 per cent CI (too small to see). (C) Map illustrating the geographical location of ∼2,000 ethno-linguistic groups in Africa (modified from Felix and Meur 2001). Key in Supplementary Table S3. (D). Map showing the ethnic diversity (Alesina et al. 2003) within Sub-Saharan Africa (orange shaded background) overlaid with pie charts demonstrating HIV-1 subtype and CRF diversity within each country (Foley et al. 2018). Missing countries (dark grey) lacked either HIV-1 or ethnic fractionalization data. The pie charts are scaled according to the number of unique patient HIV-1 sequences; a not scaled version is shown in Supplementary Fig. S3; complete key in Supplementary Table S4. (E) Map illustrating the current location of the five major language families within Africa with predominant groups indicated (modified from Felix and Meur 2001); complete key in Supplementary Table S5.

Next, we examined the relationship between the actual combined measure of ethnic diversity (Alesina et al. 2003) and HIV-1 p24Gag diversity using all HIV-1 p24Gag sequences in the HIV database regardless of subtype (Foley et al. 2018). We observed a highly statistically significant relationship between ethnic diversity and p24Gag diversity (*F*_1,13_ = 15.53, *P* = 0.0017; Fig. 2A). To examine if the combined measure of ethnic diversity also affected HIV-1 subtype diversity, we counted the number of subtypes, URFs and CRFs in each country using the HIV database sequences and allocation of subtype data (*n* = 36,044) (Foley et al. 2018). The total number of subtypes, URFs and CRFs per country was plotted against the ethnic diversity for each country weighted by the number of sequences per country and examined using linear regression. Similar to the result for HIV-1 p24Gag diversity, a significant relationship was found between ethnic diversity and subtype diversity (*P* < 2 × 10^−5^; Fig. 2B).

To further test robustness, we excluded Cameroon and the DRC where HIV-1 first circulated (Worobey et al. 2008; Faria et al. 2014), which still resulted in significant relationships (ethnic diversity versus HIV-1 p24*gag* variability (*P* = 0.0134) and subtype diversity (*P* < 2 × 10^−5^)) indicating that the timing of the epidemic in each African country was not the sole determinant of HIV-1 diversification. Collectively, these results suggest that the association between a country’s HIV-1 diversity and its ethnic diversity might be driven by the granular adaptation of HIV-1 to the HLA variants common in particular ethnic groups as countries with more ethnic groups end up with greater HIV-1 variation.

Geographically, the highest concentration of ethno-linguistic (Felix and Meur 2001), ethnic (Alesina et al. 2003) and HIV-1 subtype diversity was found along the equator, with, for example, ∼110 and ∼370 ethno-linguistic groups within Cameroon and the DRC, respectively (Felix and Meur 2001; Fig. 2C and D). As ethnic differences typically correlate with language differences in Africa (Tishkoff et al. 2009; Busby et al. 2016, 2017), we examined if HIV-1 subtypes clustered within specific language families or sub-groups. Most African ethno-linguistic groups belong to one of five language families (Niger-Congo, Nilo-Saharan, Afro-Asiatic, Austronesian, and Khoe, Fig. 2E), which each can be subdivided into several language groups, for example, the Niger-Congo family includes Bantu-speaking people that account for approximately one-third of all Sub-Saharan Africans (Patin et al. 2017) and non-Bantu-speaking people in West Africa.

We observed more similar HIV-1 subtype patterns in related ethno-linguistic groups (Tishkoff et al. 2009; Busby et al. 2016, 2017; Fig. 2D and E). Notably, HIV-C predominated in countries with the highest percentage of Bantu-speaking people (Patin et al. 2017). One exception was the many HIV-1 subtypes found in the DRC where adaptive introgression of HLA from rainforest hunter-gatherers during the Bantu expansion ∼3,000 years ago significantly increased the HLA variability of western and central Bantu-speaking people relative to that of southern Bantu-speaking people (Patin et al. 2017; Foley et al. 2018). Moreover, HIV-C was only introduced into the Horn of Africa around 1980–85 (Simmons et al. 2009), and has since, for example, in Ethiopia evolved into two distinct Ethiopian HIV-C lineages (Amogne et al. 2016). We explored whether countries which had at least 50 per cent Bantu First Language Speakers were more likely to have at least 50 per cent subtype C in their HIV-infected population. This analysis included all African countries regardless of epidemiological history and demonstrated a link between high levels of Bantu language speaking inhabitants and high levels of HIV-C (Fisher’s test, *P* = 0.037). Niger-Congo non-Bantu language groups reside in West Africa where CRF_02AG and HIV-G predominate, and HIV-C is rare. Nilo-Saharan, Afro-Asiatic and Bantu language groups predominate in East Africa where HIV-A, -D, and -C are frequently found (Fig. 2D and E).

Together with the explosion of HIV-1 subtypes in the 1960s (Faria et al. 2014), our results suggest that ethnic diversity, and hence HLA diversity, was a significant driver of HIV-1 diversification, and that neutral evolution and/or differences in the timing of local African HIV-1 epidemics was not solely responsible for the creation of HIV-1 subtypes as suggested previously (Pybus and Rambaut 2009; Peeters et al. 2013).

### 2.2 Spatio-ethnic HLA profiles associated with distinct HIV-1 subtypes

We next asked which HLA variants contributed most to the variance–covariance structure of the worldwide HIV-infected patient population by performing a PCA on all publicly available HLA Class I data from patients infected with HIV-B (*n* = 318) or HIV-C (*n* = 704; Foley et al. 2018). Because of the history of HIV-1 research, similar data on patients infected with other subtypes are rare, and most African data derive from South Africa, especially the KwaZulu-Natal province (Foley et al. 2018). We assumed that the distribution of HLA variants and HLA haplotypes (a series of HLA genes on one chromosome that is found together more often than expected due to linkage disequilibrium (LD)) in geographically dispersed populations reflected the sum of past infectious challenges and adaptive introgression from archaic humans (Doherty and Zinkernagel 1975; Segurel and Quintana-Murci 2014; Quintana-Murci 2019). For each member of the population, we proposed that their genetic ‘HLA profile’ abstractly could be considered as a point in the population’s multidimensional HLA profile space. A novel pathogen such as HIV-1 would rapidly spread and evolve within this HLA space. The space itself would only change slowly due to the added selective effect of HIV-1 infection on HLA frequencies and largely outside of our current observation time.

**Figure 3.**
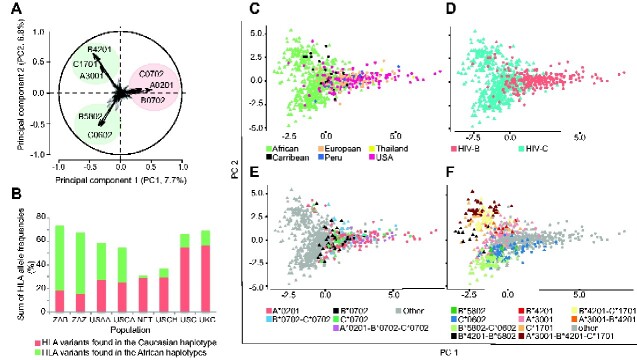
HLA PCA results coloured by country/region, HIV-subtype, and HLA-allele variants. (A) Loading plot of the direction vectors associated with each HLA type. The closer the value is to 1 (or −1) the greater the effect of the component on the variable. A patient’s position in (C–F) is obtained by summing the values for the patient’s HLA-alleles. HLA variants within African (green circle) and Caucasian (red circle) haplotypes are indicated. (B) The combined HLA-allele frequency of the individual HLA variants found in the African haplotypes (green) and the Caucasian haplotype (red) in eight populations: South African Black (ZAB), South African Zulu (ZAZ), US African American (USAA), US Caribbean (USCA), North-East Thai (NET), US Chinese (USCH), US Caucasian (USC), and, UK Caucasian (UKC). Individual HLA-allele frequencies are reported in Supplementary Table S6. Scatter plot of HLA PC1 and PC2 coloured according to: (C) geographical origin of the patient, (D) whether the patient was infected by HIV-B (red circle) or HIV-C (turquoise triangle), (E) the position of Caucasian, and (F) of predominately African HLA variants that explained the majority of variation in PCs 1 and 2.

The individual HLA variants which contributed most to the first two PCs clustered in three groups, reflecting three common haplotypes (Fig. 3A): one group containing Eurasian variants (HLA-A*02:01, HLA-B*07:02, and HLA-C*07:02) and two groups of HLA variants more common in Africa (HLA-B*58:02, HLA-C*06:02, and HLA-A*30:01, HLA-B*42:01, HLA-C*17:01; Fig. 3B and Supplementary Table S6). The first two PCs explained 14.5 per cent of the variance and the first 12 explained 50 per cent. PC1 revealed four features: (i) the HLA separation correlated with the geographical origin of the patients with Africans and Eurasians at the extremes (Fig. 3C), (ii) the Caribbean patients were near the centre, reflecting the history of admixture in that population (Fig. 3C;  Benn-Torres et al. 2008), (iii) the separation correlated with the distribution of the patients’ HIV-1 subtypes (Fig. 3D), and, (iv) the three HLA variants that contributed most to the separation were all inherited by Eurasians from Neanderthals and Denisovans (Abi-Rached et al. 2011) with HLA-A*02:01 explaining most of the variance (Fig. 3E).

PC2 robustly distinguished between two common Southern African HLA haplotypes with opposing effects on HIV-1 disease progression; HLA-B*58:02-HLA-C*06:02, linked to fast progression (found in ∼20% of the population), and HLA-A30:01-HLA-B*42:01-HLA-C*17:01, linked with slow progression through the accumulation of fitness-reducing CTL escape-mutations (found in ∼13%; (Goulder and Walker 2012; Gonzalez-Galarza et al. 2015; Fig. 3F). In Zambia, HLA-B*42:01 is also linked to a faster HIV-1 acquisition (allele frequency ∼30%) (Song et al. 2011; Gonzalez-Galarza et al. 2015). We calculated a haplotype score, which started at zero and was incremented or reduced by one for each allele in these two haplotypes, revealing the near-complete stratification of PC2 (Supplementary Fig. S4). Although these HLA variants can occur in other combinations, the LD is almost complete in Bantu-speaking South Africans and other Southern Bantu-speaking people in countries with the highest levels of HIV-C infection (Fig. 3D, F, and Supplementary Fig. S5; Paximadis et al. 2012; Gonzalez-Galarza et al. 2015).

These results suggest that the HLA variants that contributed most to the variance–covariance structure within the HIV-1 patient population worldwide originated from interbreeding between the ancestors to modern Eurasians and Neanderthals and Denisovans. An African HIV-1 lineage imported to Eurasia would, therefore, circulate in a partly new HLA profile space. Within Africa, we observed that the HLA variants that explained most of the variance were part of common haplotypes, which are associated with opposing HIV-1 disease progression patterns and facilitated HIV-1 acquisition in some regions.

### 2.3 Distinct ethnic HLA variants affect HIV-B and -C evolution

**Figure 4.**
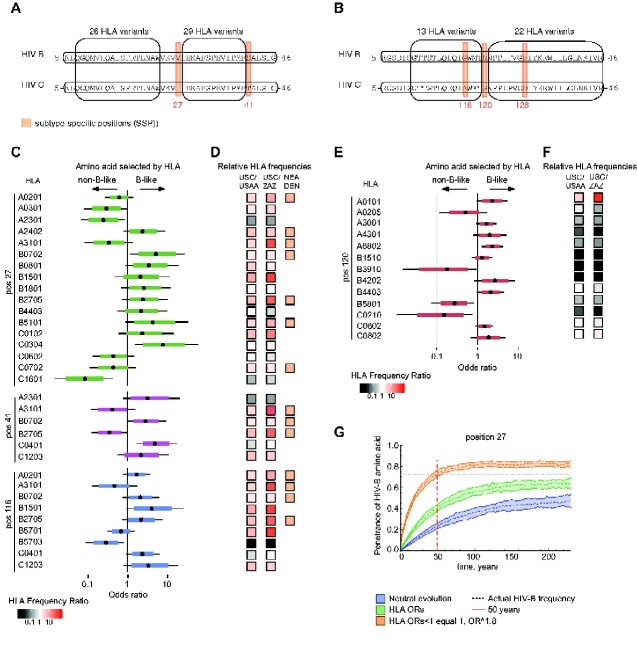
HLA-associated selective pressure on HIV-B and HIV-C. (A and B) The two conserved p24Gag HIV-1 regions. Ellipses indicate epitope-clusters with the approximate number of presenting HLA variants noted above. Orange rectangles signify subtype-specific positions (Supplementary Fig. S1). (C) HIV-B: The posterior probability distributions on HLA-associated ORs; ORs greater, or less than one, represent the belief that an HLA variant is associated with the HIV-B or non-HIV-B (i.e. African) consensus amino acid in that position, respectively. The central point represents the median, the coloured region is the 80 per cent Bayesian credible interval (BCI), and the whiskers represent the 95 per cent BCI. (D) The relative HLA-allele frequencies in US Caucasians (USC) compared with African-Americans (USAA; left hand column), and versus South African Zulus (ZAZ) (a Bantu-speaking people; middle column). HLA variants identified in DNA samples from three Neanderthals (NEA) and one Denisovan (DEN; Abi-Rached et al. 2011) are indicated (orange squares). (E) HIV-C: The posterior probability distributions on HLA-associated ORs. As in (C). (F) As in (D), none of these HLA variants were identified in Neanderthals or the Denisovan. (G) An agent-based model of neutral and HLA-mediated selection effects on HIV-C p24Gag, Position 27. The effects of neutral (blue) and HLA-mediated selection using the estimated HIV-B HLA ORs (green), and the increased HLA ORs for HIV-B-likeness (orange) are shown.

To estimate the selective pressure of individual HLA Class I variants on the diversification of subtype-specific amino acids in a population, we analysed all p24Gag HIV-B and -C sequences linked to an HLA profile (Foley et al. 2018) using a Bayesian phylogenetic HLA selection model. The analyses were performed on each subtype separately. We focussed on five subtype-specific positions (27, 41, 116, 120, and 128) situated within the two conserved p24Gag regions that dominate the clinically effective HIV-specific CTL response in patients infected by either HIV-B or -C (Goulder and Walker 2012; Fig. 4A and B and Supplementary Fig. S1). Because each subtype-specific position is independently influenced by the phylogeny (Supplementary Figs S6 and S7), the model was corrected for phylogenetic co-variation using the BEAST-derived phylogenetic variance–covariance matrix for each subtype-specific position. This correction adjusted for potential viral population structure and neutral evolution. Because some subtype-specific positions vary between subtypes, but only have limited variation within the given subtype (position 128 in HIV-B, and positions 27, 41, 116, and 128 in HIV-C), or too much variation for these analyses given our dataset (position 120, HIV-B; Supplementary Fig. S1), HLA associations could only be robustly determined for three positions in HIV-B and one in HIV-C. At the HIV-B positions 27, 41, and 116, selection typically favours one of two amino acids, and at position 120 in HIV-C, one of three (Supplementary Fig. S1). The total HLA pressure on each subtype-specific position could be considered to be the joint pressure exerted by all the HLA variants able to present epitopes encompassing (Tenzer et al. 2014) or overlapping that subtype-specific position (Foley et al. 2018; Fig. 4A and B), and by HLA variants associated through structural codon-co-variation (Carlson et al. 2008; Crawford et al. 2011). Significant statistical links between an HLA variant and selection for either HIV-B or non-HIV-B-like amino acids at the four subtype-specific positions were expressed as odds ratios (ORs).

Overall, ∼50 per cent of the Caucasian/Eurasian HLA A, B, and C variants that shaped HIV-B evolution originated from Neanderthals and/or Denisovans, which is similar to the frequencies of these variants in people of European descent (Abi-Rached et al. 2011; Fig. 4C and D). A minority of HLA variants appeared to be footprints of the on-going HIV-B epidemic in the Caribbean and in African-Americans (e.g. HLA A*23:01, HLA B*57:03, and HLA C*16:01; Fig. 4D). Although most HLA variants selected for HIV-B-like subtype-specific amino acids, some selected for non-B likeness (e.g. HLA C*16:01 (Position 27) and HLA A*31:01 (Position 41)). These results are in line with previous analyses of intra-host adaptation of HIV in (Tenzer et al. 2014), which demonstrated that the consensus HIV-B (or HIV-C) sequence was often observed in carriers of common HLA variants whereas carriers of rare HLA variants frequently selected for non-consensus amino acids. The data are consistent with the variation within our patients (illustrated in Supplementary Fig. S8) and in HIV-B sequences worldwide (Foley et al. 2018). In contrast, HIV-C evolution was shaped by African HLA variants common in Bantu-speaking people and one ancient HLA variant (HLA-A*01:01) found in parts of Africa (Abi-Rached et al. 2011; Fig. 4E and F), consistent with the spread of the HIV-C epidemic (Gonzalez-Galarza et al. 2015).

Our estimated HLA ORs (Fig. 4C and E) do not reflect the HLA selection pressure acting on the HIV-B-precursor virus during the first 30–40 years of the HIV-1 epidemic outside of Africa, because >80 per cent of our data were from 2001 or later (Foley et al. 2018), and because the epidemiological demographics have changed from predominately Caucasian until the mid-nineties to one with mixed ethnicities (Worobey et al. 2016).

To examine how the current HLA ORs and neutral selection would affect HIV-B evolution we used an agent-based epidemic model. As the first HIV-1 lineage in Haiti is unknown (Rambaut et al. 2004), we chose to test an HIV-C-like virus, which differs from HIV-B at subtype-specific positions 27, 41 and 116 (Supplementary Fig. S1). We found that neutral evolution of HIV-C would take ∼250 years to approach a 50 per cent HIV-B subtype-specific amino acid frequency (position 27, Fig. 4G, blue line; positions 41 and 116, Supplementary Fig. S9), whereas HLA-mediated selection would reach this threshold more than twice as fast (Fig. 4G, green line). This result demonstrates that the estimated HLA ORs are not only statistically significant but also biologically relevant.

Next, we tried to imitate the HLA selective pressure acting on the HIV-B precursor early in the epidemic. We ignored the selective pressure from African HLA variants and modelled how much the HLA ORs selecting for HIV-B-likeness should be increased to reach the current consensus subtype-specific amino acid frequencies within 50–55 years, a time span similar to that of HIV-B evolution (Worobey et al. 2016). We found that the HLA ORs had to be raised by a factor of 1.8–2.5 depending on subtype-specific position (Position 27, Fig. 4G, orange line; Positions 41 and 116, Supplementary Fig. S9). This result suggests that the initial HLA-mediated selective pressure on the HIV-B-precursor was either stronger than our estimates and/or that we are missing the effect of some HLA variants due to viral adaptation before 2001. Alternatively, or additionally, some of the subtype-specific amino acids in the HIV-B-precursor might have been more similar to HIV-B than HIV-C, and/or structural co-evolutionary effects (Carlson et al. 2008; Crawford et al. 2011) might be stronger than expected.

Collectively, our analyses reveal that HIV-B and HIV-C circulate in populations with distinct, and partly non-overlapping, HLA profiles and that the difference in population-specific HLA-mediated selective pressures affected HIV-1 evolution.

### 2.4 The emergence of an African American-adapted HIV-B

**Figure 5.**
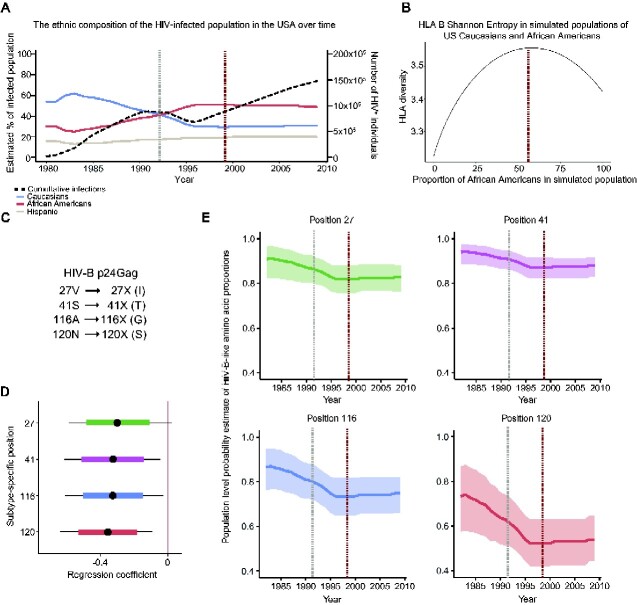
HIV-B adaptation to the proportion of African Americans in the US HIV-infected population over time. (A) The estimated ethnic composition of the HIV-infected population in the USA over time. The grey vertical line indicates the time of equal proportions of Caucasians and African Americans, and the dark red vertical line when the proportion of African Americans had stabilized. (B) Simulation of the relationship between the proportion of African Americans in a mixed African American and US Caucasian population and the HLA B Shannon entropy (HLA B diversity) of those mixed populations. Red dotted line highlights the maximum HLA diversity (all HLA simulations and HLA frequencies in Supplementary Fig. S10). (C) Overview of the changing subtype-specific positions; HIV-C subtype-specific amino acids are shown in parentheses and represent the most common change. (D) Posterior distributions over the regression coefficients of our model. The central point represents the median, the coloured region is the 80 per cent BCI, and the whiskers represent the 95 per cent BCI. The 95 per cent BCI exclude 0 for Positions 41, 116, and 120, whereas the 95 per cent BCI for Position 27 slightly overlap zero. (E) Population-level estimates of the probability a subtype-specific position is occupied by an HIV-B subtype-specific amino acid as explained by the fraction of African Americans plotted against time (with the 95% BCI).

If the subtype-specific differences between HIV-B and HIV-C are the result of different HLA-allele frequencies between Eurasian and African populations, changes in the ethnic demographics of an HIV-infected population should affect the selective pressure at the subtype-specific positions. To investigate this hypothesis, we examined the HIV-1 epidemic in the USA. HIV-B was likely introduced in the 1970s (Worobey et al. 2016) and initially circulated primarily amongst Caucasian MSM. However, the epidemic gradually spread to other groups, and, regardless of sexual orientation, African and Hispanic Americans have increasingly been disproportionally affected (Fig. 5A). A complex set of socioeconomic factors drive risk to these groups, including discrimination, stigma, poverty, lack of access to care, and cultural differences combined with in-group sexual bias (Centers for Disease Control and Prevention 2020, www.cdc.gov). Currently, African and Hispanic Americans account for ∼42 and 20 per cent of all new infections despite making up only about 13 and 16 per cent of the US population, respectively (Hall et al. 2008; Prejean et al. 2011).

Based on our previous HLA diversity analysis (Fig. 1), we proposed that the increased fraction of non-Caucasians in the US HIV-infected population would increase its HLA diversity. To investigate, we performed simulations using the HLA A, B, and C frequency data for US Caucasians and African Americans reported by Gragert et al. (2013). Briefly, we created mixed populations of the two groups and calculated the HLA Shannon entropy (diversity) of the simulated mixed populations (HLA B is shown in Fig. 5B, all HLA simulations and plots of HLA frequencies are shown in Supplementary Fig. S10). The results demonstrated that the HLA B and HLA C diversity peaked when the two groups were present in approximately equal proportions, whereas the HLA A diversity continuously increased as the proportion of African Americans grew. These differences were due to the relatively lower diversity of US Caucasian HLA A alleles and the more similar diversity of HLA B and C alleles between the groups (Supplementary Fig. S10). For simplicity, we did not add HLA data from US Hispanics, which are more like those of US Caucasians. Adding these would only increase the overall diversity.

Because 73–78 per cent of the African American genetic heritage originates from Africa (∼30% from Bantu-speaking people and ∼70% from regions of West Africa; Patin et al. 2017), we hypothesized that the HIV-B subtype-specific amino acids would evolve towards amino acids more commonly found in African HIV-1 strains than in HIV-B (Fig. 5C). We estimated the ethnic demographics of the HIV-infected population in the USA from (Hall et al. 2008) and (Prejean et al. 2011) and used that demographic data to calculate the frequency of African Americans in the US HIV-infected population over time (Supplementary Fig. S11; Gonzalez-Galarza et al. 2015).

We compiled all dated HIV-B p24Gag sequences associated with a patient ID (to prevent oversampling sequences from single patients) and used these in a Hierarchical Bayesian multi-level model that incorporated phylogenetic information from BEAST to prevent phylogenetic relationships from biasing the analysis, thereby correcting for viral population structure and neutral evolution (Supplementary Fig. S12). The posterior distributions over the regression coefficients of the phylogenetic model robustly demonstrated that the probability that the 41, 116, and 120 subtype-specific position was occupied by an HIV-B subtype-specific amino acid was inversely associated with the frequency of African HLA variants in the infected population (Fig. 5D). Although the pattern was similar for Position 27 and the 80 per centBCI excluded zero, the 95 per cent BCI did not quite exclude zero.

Collectively, our analyses demonstrated that the subtype-specific positions in HIV-B over the last 20 years in the USA have evolved away from the original HIV-B consensus sequence and increasingly incorporate amino acids found in African HIV-1 subtypes (Fig. 5E, Supplementary Fig. S12, and Table S7). These results show that HIV-1 diversification is an on-going process that can be influenced by changes in the ethnic composition of the HIV-infected population within a country. As African Americans continue to suffer a disproportionate number of HIV-infections, HIV-B is likely to continue to adapt to the HLA variants common in this group.

## 3. Discussion

In this study, we found that HLA Class I allelic frequency differences and archaic HLA Class I variants combine to create population-specific HLA-associated selective pressures that uniquely shape HIV-1 p24Gag variation and subtype diversification. This observation suggested that these population-specific HLA-associated selective pressures might also shape the evolution of other novel pathogens in emerging epidemics, for example, Zika virus and Severe acute respiratory syndrome coronavirus 2 (SARS-CoV-2), and might be partly responsible for the subtype diversification of viruses that have a longstanding infection history in humans (e.g. Hepatitis B, Hepatitis C, and Morbilli). Similarly, these population-specific selective pressures could affect the evolution of old pathogens when these spread into previously naïve populations in new geographical locations due to human activities, and/or climate change (e.g. dengue virus and West Nile virus). This granular adaptation of pathogens to a population-specific HLA composition mirrors the already accepted granular adaptation of human populations to different combinations of infectious challenges (Doherty and Zinkernagel 1975).

The adaptive introgression of Neanderthal and Denisovan HLA variants in Eurasians, and of HLA B*73:01 (a unique major histocompatibility complex (MHC)-BII allele more closely related to chimpanzee and gorilla MHC-B alleles than to other modern human B alleles; Abi-Rached et al. 2011), and more recently of rainforest hunter-gatherers HLA variants in Bantu-speaking people (Patin et al. 2017) collectively suggest that introgression of pre-adapted, advantageous HLA variants might have occurred throughout hominid evolution and modern human history as invading populations mixed with adapted locals. Such adaptive introgression events are likely not limited to primate evolution. The repeated introgression of immune response-related genes would expose locally established pathogens to a continuous selective pressure for longer than the length of time that the most recently immigrated host lineage has been in a place.

In Africa, we found significant positive relationships between ethnic diversity and HIV-1 p24Gag sequence and subtype diversity, respectively. These robust associations suggested that ethnic diversity, and by extension HLA diversity and differences in HLA-associated selective pressures, helps drive HIV-1 evolution and might result in subtype diversification. It will be important to confirm and extend the findings reported here across other antigenic regions of HIV-1. The spread and adaptation of HIV-1 to different ethnic groups might initially have been facilitated by, for example, a combination of the socioeconomic and cultural changes induced by the colonization of the DRC in the early 1900s, for example, the rise of cities, extended transport networks, and centralized camps for treatment of sleeping sickness (Worobey et al. 2008; Faria et al. 2014).

Our results, and previous experimental work (Frahm et al. 2006; Tenzer et al. 2009, 2014), suggest that the local HIV-1 consensus sequence represent the most common adaptions to escape effective immune responses in the majority of people in the population in which it circulates. Consequently, the local HIV-1 consensus sequence is the least likely sequence combination to evoke effective immune responses upon transmission. This proposal is in line with reports that the virus closest to the consensus sequence in a population is most readily transmitted (Carlson et al. 2014; Song et al. 2011). Because the transmitted virus only needs to be present in small amounts in the infectious dose (Keele et al. 2008), these observations together might help the understanding of why the HIV-1 evolutionary rate at the within-host compared with the population scale is ∼5-fold faster (Raghwani et al. 2018). The predominant viral variants in the host are typically those best adapted to escape that particular host’s specific immune responses, while those viruses that are most likely to be transmitted are those most like the local consensus sequence. These ‘consensus-like’ sequences are likely to be found as a minority population and to be more closely related to the initially infecting viral variants, compatible with both the ‘store and retrieve’ and ‘adapt and revert’ scenarios suggested by Raghwani et al. (2018).

Between 1962 and 1970, HIV-1 spread beyond Africa as a Haitian aid-worker returned from the DRC (Gilbert et al. 2007). Whether this person was infected by HIV-B or a Subtype-B/D ancestor lineage is unknown, but the latter is likely given the lack of reports of indigenous HIV-B epidemics in Africa (van Harmelen et al. 1997; Hemelaar et al. 2011; Foley et al. 2018). The Haitian HIV-B epidemic is characterized by being more genetically diverse than elsewhere, and the predominant viral lineages are distinct from the pandemic HIV-B lineage (Gilbert et al. 2007). Despite ∼70,000 American tourists visiting Haiti every year throughout the 1970s, and a rapidly developing hetero- and homosexual sex industry (Giraud 2010), it took ∼10–12 years before HIV-B spread within the USA to a sufficient extent for the epidemic to be detected (Gilbert et al. 2007; Worobey et al. 2016). It is also noteworthy that no HIV-1 outbreak was observed in Belgium following the return of the colonialists when Congo achieved independence in 1960. The causes of this lack of spread or these interludes are debateable and likely multiple, partly dissimilar, and complex. However, we propose that one shared contributing factor could be lower transmissibility of African HIV-1 lineages to and between Europeans, perhaps because these HIV-1 lineages were not pre-adapted to escape immune responses associated with HLA variants common in Caucasians. This hypothesis is indirectly supported by reports of; (i) faster disease progression in HLA-matched recipients of pre-adapted HIV-1 (Carlson et al. 2016), (ii) less polyfunctional and more narrow CTL responses and higher viral loads in infected vaccine recipients with higher HLA I adaptation to the Gag vaccine insert (Boppana et al. 2019), and (iii) studies in Zambia demonstrating an association between specific, common HLA variants and faster HIV-1 acquisition (Song et al. 2011) and a more readily transmission of the predominant HIV-C consensus sequence (Carlson et al. 2014). In combination, these observations suggest that the increasing HIV-B adaptation to African-Americans, and the on-going high infection rates, could result in faster transmission and disease progression in this group.

Our results, and the many HIV-1 vaccine trial failures (Barouch and Korber 2010), suggest that the starting point for HIV-1 vaccine design should be the HLA composition of the target population, not the HIV-1 subtype infecting that population as in, for example, the unsuccessful Step and Phambili trials (Corey, McElrath, and Kublin 2009), because the circulating subtype will have adapted to avoid evoking strong immune responses in the majority of the population (Tenzer et al. 2009, 2014). One vaccine design option would be to select in-natural-infection subdominant epitope sequences from vulnerable parts of HIV-1 (Dahirel et al. 2011) that are presented by HLA variants common in the target population and embed these in artificial contexts that promote high epitope production. The resulting abundant epitope production will help prime anti-viral CTL responses (Faroudi et al. 2003), while even limited production of these epitopes after HIV-1 infection will allow the primed CTLs to recognize and kill infected cells (Purbhoo et al. 2004).

## 4. Materials and methods

### 4.1 Simulation of the relationship between ethnic diversity and HLA diversity

To investigate the relationship between ethnic diversity and HLA diversity we compiled HLA data from all available African populations/ethnic groups that had been HLA typed to a ‘Gold standard’ (i.e. all HLA types were reported as four-digit HLA variants) (*n* = 8; detailed in Supplementary Table S1). Although three of the seven African populations came from Kenya, they belonged to distinct ethnic groups and were, therefore, all included. The eight populations were mixed in arbitrary proportions to simulate the mixture of ethnic groups within different fictive African ‘countries’. The proportions were assigned as follows: k random draws were taken from a uniform distribution. These were turned into proportions by dividing each random draw by the sum of all random draws. HLA types were mixed proportionally into each simulated ‘country’, and Shannon entropies were calculated both for the simulated ethnic diversity and the simulated HLA diversity. Linear regressions were performed of the Shannon entropy of the HLA diversity calculated from these mixes versus the population Shannon entropy calculated from the simulated mix. Statistically significant results were obtained for linear regression analyses of simulated countries consisting of n ‘ethnic groups’ and for all simulated countries (not shown).

### 4.2 Shannon entropy analyses of African HIV-1 p24Gag diversity

To calculate the Shannon entropy (diversity) of HIV-1 group M p24Gag for each country we searched the LANL HIV database (Foley et al. 2018) for one non-problematic HIV-1 p24Gag sequence per patient from Sub-Saharan Africa (each sequence was ≥ 684 nucleic acids in length (∼99% of the HXB_2_ p24 sequence (GenBank K03455))) using the integer patient ID tool. Subsequently, we removed all sequences from HIV-1 Groups N, O, and P, and URFs and CRFs thereof, as well as sequences without a unique patient ID to avoid oversampling individual patients. We screened the remaining sequences for those originating from uncultured peripheral blood mononuclear cells, and those originated from cultured virus by examining all paper references. All sequences originating from cultured virus were discarded because culturing likely obscures the sequence footprints of the intra-host immune response. The remaining 2,274 sequences were aligned using the LANL HIV Align program, after which the sequences were stratified by country. Minor manual corrections were made to each country’s alignment as needed, and the mean amino acid Shannon Entropy of each of these alignments was calculated using the R (version 3.4.3) package bio3d (version 2.3-3).

### 4.3 Shannon entropy analysis of ethnic diversity

As a proxy for each country’s HLA diversity, we used ‘ethnic diversity’, which was based on the combined measure of ethnic, linguistic, cultural, and religious fractionalization defined by Alesina et al. (2003). This combined measurement is based on data from several sources, including Encyclopaedia Britannica, The CIA World Factbook, Minority Rights Group International, and (Levinson 1998). The ethnic diversity for each country was estimated by calculating the Shannon entropy for the ethnic proportions data in (Alesina et al. 2003) as follows:

$$H=\;-\sum_{i=1}^{n}p_{i}\log p_{i}$$

where *n* indicates the number of ethnicities recorded with the database, and $p_{i}$ is the proportion of ethnicity *i* within the country’s population.

### 4.4 Examination of ethnic versus HIV-1 p24Gag diversity

Ethnic diversity was plotted against the mean HIV-1 p24Gag amino acid entropy, weighted by the number of p24Gag sequences per country, using ggplot2 (version 2.2.1). The size of each dot was scaled logarithmically by the number of sequences to indicate graphically how this weighting was occurring. A weighted linear model was fit in R (version 3.4.3) to assess statistical significance.

### 4.5 African HIV-1 sequences and subtype analysis

We searched the LANL HIV database (Foley et al. 2018) for all non-problematic HIV-1 sequences originating in Africa (176,071 sequences). HIV-1 from Groups N, O, and P, and URFs and CRFs thereof were removed, and all HIV-1 group M pure subtypes, URFs and CRFs were filtered for one sequence per patient according to the integer patient ID (Foley et al. 2018). Sequences were then filtered for those originating in Sub-Saharan African (as defined by the United Nations Statistics Division, not LANL) yielding 36,044 sequences. From these, the number of subtypes, URFs, and CRFs per country and the number of sequences of each subtype, URF, and CRF per country was calculated. The total number of subtypes, URFs and CRFs per country was plotted against the ethnic diversity for each country weighted by the number of sequences per country using ggplot2 (version 2.2.1). The size of each point was scaled logarithmically by the number of sequences. A weighted generalized linear model was fit using R (version 3.4.3) to determine the significance. We generated a map with pie charts showing the relative amounts of each subtype in the LANL database for each country, using Tableau 10.4 (Fig. 2D; a non-weighted version is shown in Supplementary Fig. S3). The ethnic diversity for each country was also displayed on this map. We generated a map showing the African ethno-linguistic groups (Fig. 2E). For each African country we obtained the proportion of First Language Users of Bantu languages in the general population from Ethnologue (2020; www.ethnologue.com). For ease of analysis we dichotomized this variable using 50 per cent as a threshold value. Similarly, we divided the African countries into two groups by the prevalence of HIV-C in their HIV-infected population.

### 4.6 Worldwide HIV-infected patient information

To study potential HLA driven selection on HIV subtype-specific motifs, we searched the LANL HIV database (Foley et al. 2018) for all HLA typed HIV-1-infected patients from which HIV-1 p24Gag had been sequenced. For each patient, we obtained the majority rule consensus for five Gag subtype-specific positions (Positions 27, 41, 116, 120, and 128, Supplementary Fig. S1). The LANL HLA annotations were manually curated to ensure a consistent HLA nomenclature (e.g. ‘B2705’, ‘B*27:05’, and ‘HLA-B*27:05’ were all rewritten as ‘B2705’). We used HLA annotations with four or more digits to generate an HLA presence–absence matrix to avoid pooling HLA variants with different effects on HIV evolution (e.g. HLA-B*35-Py/Px and HLA-B*58:01/02 (Goulder and Watkins 2008; Goulder and Walker 2012)). However, this matrix was extended by two-digit HLA annotations if the four-digit HLA type could be inferred based on (i) the patients HLA variants (typical for either Caucasians or African Americans), or (ii) the escape mutation pattern in their HIV-1 sequences (see Supplementary Table S8 for patient ID). For example, patients with HLA-B*27:05 often target the KK10 epitope, and the R264K, R264T, or L268M substitutions are often present within the epitope, but this is not the case in patients with HLA-B*27:02. In total, four-digit HLA types were inferred for 23 patients with HLA-B*27 (Goulder et al. 2001; Goulder and Watkins 2008; Goulder and Walker 2012). Likewise, 29 B*57 patients were reclassified as B*57:01 based on the associated paper reference (Migueles et al. 2003) and two patients were classified as B*57:01 carriers based on their other HLA types and cohort demographics (Goulder and Watkins 2008; Goulder and Walker 2012). We reassigned six American patients with HLA type B*35 to B*35:01 in African-American patients, as the frequency of HLA B*35:01 in this ethnic group is far higher than that of HLA B*35:02 or HLA B*35:03 (Gonzalez-Galarza et al. 2015). After assessing all the publicly available data, we limited ourselves to HIV-B (*n* = 318) and HIV-C (*n* = 704), the only subtypes for which there were enough patients to perform our analyses.

### 4.7 HLA presence–absence matrix

We generated a binary HLA presence–absence matrix; the rows represented patients (*n* = 1,022), and the columns the HLA Class I variants (the total number of HLA A, B, and C variants was 78). The entries were 1 when a patient was annotated with an HLA variant in the LANL database, and 0 otherwise resulting in an HLA profile for each patient (the table is available upon request).

### 4.8 Population HLA Class I information

The HLA frequencies of all populations were derived from the HLA-allele database (Gonzalez-Galarza et al. 2015) except for the South African Zulu population. These HLA frequencies were obtained as part of the Females Rising through Education, Support, and Health project and were shared by Professor Bruce Walker, Harvard University, and Professor Mary Carrington, Frederick National Laboratory for Cancer Research.

### 4.9 Exploratory PCA

To investigate the variance–covariance structure of the HLA presence–absence matrix, we performed a PCA (Menozzi, Piazza, and Cavalli-Sforza 1978) and created a scatterplot of the first two PC scores (Fig. 3 and Supplementary Fig. S4). Briefly, PCA uses an orthogonal transformation to convert the variables in the HLA presence–absence matrix into a smaller set of linear combinations called PCs and determines the minimum number of factors that will account for the maximum variance in the data. The first PC accounts for as much of the variability in the data as possible, and each succeeding PC accounts for as much of the remaining variability as possible.

### 4.10 Accounting for phylogenetic variance

In our statistical model, we explicitly accounted for the phylogenetic covariance between individuals infected by closely related viruses by including a parameter ($u_{ij})$ for every patient (*i*) and amino acid position (*j*) combination. Every column in this matrix (representing the phylogenetic effect for one subtype-specific position) was modelled by a multivariate normal distribution over patients using a phylogenetically informed covariance matrix, thereby representing the similarity between patients through the relatedness of their viruses. This phylogenetic correction was necessary because, for example, antenatal transmission could lead to closely related viruses (with very similar amino acids in the subtype-specific positions) in patients with similar HLA variants even in the absence of HLA-associated selection. We chose to use a separate phylogenetically informed multivariate normal for each subtype-specific position because using just a single multivariate normal (i.e. a single parameter per patient, shared over all *k* subtype-specific positions, from a phylogenetically informed multivariate normal distribution) would fail to capture the independence of each subtype-specific position.

This model can be expressed as follows:

$$\mathrm{logit}(p_{ij})=\alpha_{j}+\beta_{j,1:l_{j}}\cdot X_{i,j,1:l_{j}}+u_{ij}$$

 

$$u_{*j}∼N_{n}(0,\sigma C)$$

 

$$y_{ij}∼\mathrm{Bernoulli}(p_{ij})$$

n is the number of patients.k is the number of amino acid positions.$l_{j}$ is the number of HLA types used as explanatory variables for subtype-specific position j.

Y is a n×k matrix (with individual elements $y_{ij}$) of binaries representing whether the majority rule amino acid in a patient is the same as the HIV-B Majority Rule consensus amino acid.

X is a $n\times k\times l_{j}$ array of binaries representing the selected HLA types for the jth subtype-specific position. This ragged 3D construction is necessary because we use different sets of HLA types as explanatory variables for each amino acid; hence a separate 2D HLA matrix is needed for each position.

β is a $k\times \max(l_{j})$ matrix representing the HLA coefficients for the *k* different amino acids. Vectors $\beta_{j*}$ are not used in the model beyond index $l_{j}$; this is because ragged arrays are not available in Stan (the software we used to do Markov chain Monte Carlo (MCMC) analysis; Carpenter et al. 2017).

We define P as an n×k matrix containing the inferred probabilities $p_{ij}$ of amino acid j in patient i being B-like.

C is a covariance matrix representing genetic relatedness between the viruses infecting the patients in our sample (see below).

The parameterization $\beta_{j,1:l_{j}}\cdot X_{i,j,1:l_{j}}$ was chosen because there is little overlap between the HLA types that selected for each amino acid position. The $l_{j}$ variable is necessary because different numbers of HLA types are appropriate for the different subtype-specific positions.

Gelman’s recommendation (Gelman et al. 2008) for weakly informative priors appropriately scaled for logistic regression was adopted for the alpha and beta parameters; namely Cauchy distributions with a location parameter of 0 and a scale parameter of 2.5. The σ parameter was given a half-normal prior with a 0 mean and a standard deviation of 1. Because this analysis is highly parameterized (one $u_{ij}$ per data point), allowing σ to vary resulted in high sampling variance in σ and the $u_{ij}\;$parameters until the inverse logit function collapses to 0 or 1 for all data points, leading to instability of the σ parameter and non-convergence due to non-identifiability. To prevent this, we set σ = 1. This choice of parameter is reasonable in the context of a logit link function, which rapidly approaches 0 and 1 outside the range [−2.71, 2.71]. This method is based on a previous Bayesian model which only allowed for linear regressions (de Villemereuil et al. 2012), which we here extend to allow the fitting of generalized linear models. We do not incorporate phylogenetic uncertainty, but instead use a point-estimate of the phylogenetic correlation matrix C.

We used simulations to justify our choice for an independent phylogenetically informed multivariate Gaussian prior for each amino acid position. We simulated each amino acid separately using a Bernoulli distribution where *p* is a draw from the multivariate normal distribution using a phylogenetic variance–covariance matrix and the logistic link function; *x* was a draw from a multivariate Gaussian distribution using the same phylogenetic variance–covariance matrix. We varied the number of amino acids simulated between 1 and 9 and generated 2,000 simulated datasets for each amino acid count. We fitted a model with a single phylogenetic multivariate Gaussian distributed random effect shared between all amino acids in a patient, and a model with a separate phylogenetic multivariate Gaussian distributed prior for each amino acid. We found that the model with the shared multivariate Gaussian was prone to increase false positive rates (FPRs) and that the FPR increased with increasing numbers of simulated amino acids. The FPR in the model with a separate phylogenetical multivariate normal distribution for each amino acid was stable. Stan code is available upon request.

### 4.11 Generating the phylogeny and the phylogenetic correlation matrix

For each subtype, we built manually curated alignments with a single representative sequence per patient and used the LANL HYPERMUT tool to remove hypermutated sequences. To avoid biasing the analysis by including HLA selective effects in the correlation matrix C, which could occur since CTL responses can drive convergent evolution leading to clustering in phylogenetic trees (Matthews et al. 2009), we removed from the alignment the five subtype-specific columns we use as response variables in the multilevel model, and columns subject to CTL escape mutation known to skew phylogenetic analysis. BEAST was used to sample from the posterior distributions of phylogenies by running 10 chains for 200 million steps with an HKY+G model of nucleotide evolution, a lognormal clock, and a logistic growth coalescent tree prior. The initial portions of the chains were discarded as burn-in (50 million for HIV-B; 100 million for HIV-C, except one HIV-C chain for which we discarded 150 million). We evaluated the posterior using CODA (Plummer et al. 2006), and ensured that all effective sample sizes (ESS) for continuous parameters were larger than 200. We used TreeAnnotator to build a Maximum Clade Credibility (MCC) tree for each subtype, keeping the heights of the maximum posterior tree to prevent visualization artefacts. For every tree sampled from the posterior, we calculated a phylogenetic variance–covariance matrix (Felsenstein 1985; Freckleton, Harvey, and Pagel 2002). By averaging over all sampled trees, we estimate the posterior phylogenetic variance–covariance matrix scaled in years, which was transformed into the phylogenetic correlation matrix C.

### 4.12 Cross-validation

To avoid overfitting the high number of HLA types relative to the number of patients (HIV-B: 26 HLAs/292 patients; HIV-C: 45 HLAs/687 patients) we performed a 5-fold cross-validation procedure (Kohavi 1995). This procedure incorporated both feature selection and model fitting, which allowed us to optimize the bias-variance trade-off. Within each training set, we calculated the correlation coefficients between each subtype-specific position and all HLA types; we then constructed Bayesian models with the *l* best-correlating HLA types for every subtype-specific position and used the inferred parameters to predict the excluded validation sets. From these predictions, we calculated the area under the curve (AUC) of the receiver operator characteristic curves (ROC) values for each subtype-specific position (Fawcett 2006). We chose this metric because it is invariant to the calibration of the latent probabilities in the Bernoulli model. The training and validation sets were constructed on the patient level to avoid upwardly biasing the AUC (Saeb et al. 2016).

This procedure was repeated for every *l* until all HLA variants were included in the model. After 10 replications, to reduce cross-validation error, a LOESS (LOcally Estimated Scatterplot Smoothing) curve of the results was calculated for each subtypes-specific site. In the cross-validation model fitting procedure on the training set, all $l_{j}$ were identical. For each subtype-specific position, the $l_{j}$ associated with the highest AUC was taken as the starting point for the parsimonious model. Cross-validation was performed on HIV-B and HIV-C separately for both the patient-parameter and the phylogenetic model. The numbers of HLA variants vary at each position in Fig. 5 as they reflect the point when adding additional HLA variants ceased to improve our predictions for that particular subtype-specific position.

### 4.13 Parsimonious model

For the parsimonious models, we took the $l_{j}$ HLA types for each position which had the highest correlations with the amino acids, calculating the correlations using the entire dataset, where $l_{j}$ was the optimum number of HLA types for a subtype-specific position *j* according to the cross-validations. We then fitted a multilevel Bayesian model using these predictors and refitted it after removing any HLA types where the associated OR 80 per cent BCI failed to exclude 1. We obtained posterior distributions from the model using the Stan MCMC sampler (Carpenter et al. 2017). We ran four chains for 2,000 generations each, discarded the default 50 per cent burn-in and confirmed convergence by ESS >500 for all parameters, $\hat{R}$<1.01 and satisfactory Gelman diagnostics (Gelman and Rubin 1992; Brooks and Gelman 1998; Gelman et al. 2008). Pairs plots of the beta posteriors were examined to check for collinearity between the HLA types, which could arise if two or more included HLA types were part of a common haplotype. Pair plots of the MCMC traces were examined at every step to search for potential interactions.

### 4.14 An agent-based method to investigate HIV-1 subtype diversification

To create our model, we combined a transmission dynamics model with a model for within-host HIV-1 evolution (both described below). We examined the effect of HLA selection on HIV-1 evolution in two ways. First, we used the HLA ORs estimated from the HIV-B HLA regression dataset and evaluated how these would affect the modelled amino acid frequencies (Fig. 4G and Supplementary Fig. S9) and second, we tested how much the HLA ORs would have to be increased to result in the HIV-B amino acid frequencies observed today. To more adequately capture the dynamics of the early, primarily Caucasian, HIV-1 epidemic outside of Africa, we adjusted all HLA ORs from HLA variants common in AfricanAmericans to 1. Because only non-B like subtype-specific amino acids are present initially in our model, there would be no selective pressure against HIV-B-like amino acids as this selective pressure would originate from the CTL response against epitopes that are only processed, or processed to a greater degree, when an HIV-B-like amino acid is present in a subtype-specific position. To reflect this, we restricted all odds <1 (i.e. those which drive evolution away from subtype-B) to equal 1.

#### 4.14.1 *Model part 1: transmission dynamics*

We assumed a human population of a fixed size into which an initial infection of an HIV-C-like virus was introduced to a small subset of the population at time zero. The infection subsequently spreads stochastically through the population according to the following processes:

- Random mating between an infected person and one of the susceptible individuals (S) occurs at a rate β. This assumption implies there is no spatial structure to the community that is, it is panmictic.
- Death of a susceptible individual occurs at a rate $\mu_{S}$.
- Death of an infected individual occurs at a rate $\mu_{I}>\mu_{S}$.

To maintain a constant population size, whenever an individual died they were replaced with a susceptible individual. We assumed that all the individuals in the population were susceptible to HIV-1 before the infection was introduced. After the introduction, the population was composed of a mix of susceptible and infected individuals. In this model, individuals could not recover from their HIV-1 infection, and so the infection dynamics followed that of a stochastic ‘susceptible-infectious’ model. To simulate the dynamics we used the continuous time Gillespie algorithm (see Erban, Chapman, and Maini 2017).

#### 4.14.2 *Model part 2: within-host evolution of the HIV sequences*

We recognize that in reality, an individual is usually infected with a limited number of HIV-1 variants that are not specifically adapted to the recipient. In our simulations, however, we suppose that within-host evolution of HIV-1 is sufficiently rapid to ensure that the HIV-1 population within a patient during most of the infectious period (i.e. after ∼9 months; Karlsson et al. 2007; Goulder and Walker 2012; Abrahams et al. 2013) becomes dominated by HIV-1 that has adapted to that particular host.

We modelled the observed HIV-B and HIV-C subtype-specific sites in HIV-1 p24Gag and assumed that the specific amino acid present at each of these positions evolved in accordance with the HLA profile of the host to be either HIV-B- or HIV-C-like. When individuals in our model are ‘born’ they are endowed with an HLA profile, which is drawn from an underlying pre-specified distribution for a collection of HLA types for which we have determined the selective ORs for selecting for an HIV-B-like amino acid if HIV-1 infected. When each individual is created, they can have a maximum of two (and a minimum of zero) alleles for each of the HLA A, B, and C with associated ORs. We recognize that in real life, an individual will possess two alleles for each of the three HLA classes. In our model, if an individual has, for example, zero HLA A variants associated with HLA ORs, then this means that their two HLA Class A alleles are not amongst those we model (and have an OR of one; see below).

For a given amino acid position j we calculated the combined odds across all HLA classes belonging to the host by taking the product of the corresponding odds,

(4) $$\begin{array}{c} {\bar{\eta }}_{j}=\prod_{i=1}^{K}\eta_{ij}, \end{array}$$

where $K\in \left(3,4,5,6\right)$ is the number of HLA classes that we model, and $\eta_{ij}$ is the estimated odds for HLA class i and amino acid position j. We used an independent continuous time Markov Chain to model the evolution of each amino acid position. Specifically, we assume a transmission matrix of the form,

$$\Gamma =\left[\begin{array}{cc} \left(1-\gamma \right){\bar{\eta }}_{j} & \gamma \\ {\bar{\eta }}_{j}\gamma & \left(1-\gamma \right) \end{array}\right],$$

where γ is the mutation rate of an amino acid. The matrix is orientated so that the diagonal entries determine the rate at which an amino acid position remains at its current state (top-left corresponding to the rate of remaining in an HIV-B state, bottom-right to remaining in an HIV-C state), and the off-diagonal elements correspond to the rate of mutation. In the matrix, the left-hand column is multiplied by the estimated odds which result in neutrality if ${\bar{\eta }}_{j}=1$, a bias towards an HIV-B if ${\bar{\eta }}_{j}>1$, or a bias towards HIV-C if ${\bar{\eta }}_{j}<1$. If one individual infects a susceptible (multiple HIV inoculations within one individual are not allowed in our model) then they are given an HIV variant with a sequence that corresponds to the current sequence (i.e. the result of the evolving Markov Chain after the time since infection) within the infected person.

#### 4.14.3 *Model sequence*

In the simulations, we first created a population of N individuals, and then randomly infect an initial number $I_{0}$ of the populace with HIV-1 with one particular sequence (in our simulations variants with all amino acids in the HIV-C positions). We then iterate the following until a given time T has elapsed,

1. Determine the time step t until the next ‘event’ and the type of event (an infection, a death of a susceptible, or a death of an infected) to occur by the Gillespie algorithm.
2. Implement the event.
3. For each infected individual in the population evolve each HIV amino acid position as a continuous time Markov Chain for time t with rates determined by their HLA type.

We used the parameters shown in Table 1 in the simulations. The transmission rate at the start of the epidemic was obtained from Varghese et al. (2002).

**Table 1.** The parameters used in the agent-based agent-based epidemic model

Parameter	Interpretation	Value
n	Population size	1,000
$I_{0}$	Initial infected group size	500
T	Total time	230 years
β	Transmission rate	0.0002 per infected/ susceptible pair
$\mu_{S}$	Death rate of susceptibles	0.02 per year
$\mu_{I}$	Death rate of infected	0.1 per year
γ	Amino acid mutation rate	0.06 per year

### 4.15 HIV-infected US patient sequences and HLA information

To assess HIV-1 evolution over time in the USA, we searched the LANL HIV sequence database (Foley et al. 2018) for all dated HIV-B sequences that spanned HXB2 nucleotide coordinates 1,252–1,569 (a fragment of p24Gag) and were sampled in the USA and annotated with a patient ID (*n* = 9,729 HIV-B sequences from 437 patients). This limitation was necessary to avoid pseudo-replication through the inclusion of multiple sequences from the same patient as unique data points. In contrast to the compilation of the worldwide HIV-infected patient sequences, we also included patients without HLA information and allowed for multiple sequences to be used per patient rather than taking the majority rule amino acid.

### 4.16 Building a posterior distribution of phylogenies from US sequences

We ran 10 BEAST chains for 100 million steps using a single representative sequence per patient and a GTR+G model of nucleotide evolution, using a log-normal clock and a logistic coalescent; this has previously been shown to be appropriate for similar data (Worobey et al. 2016). To promote model convergence, we fully incorporated ambiguous sites in this model and did not treat them as missing. We removed an appropriate 20 per cent burn-in and confirmed ESS > 200 on all continuous parameters in CODA (Plummer et al. 2006). We used TreeAnnotator to calculate a MCC summary tree.

### 4.17 Bayesian model comparison with BEAST from US HIV-1 sequences

We also used the posterior distribution of trees to perform some exploratory analyses. It was not possible to use BEAST to analyse multiple sequences sampled from a patient. Instead, we limited our analysis to whichever sequence was most recently sampled from the patient, to allow the virus the most time to adapt to its host. We treated the amino acids as binary traits, which can be either positive if the amino acid in the patient’s subtype-specific position is the same as the HIV-B consensus, or negative if it is different. We fitted four models of Discrete Trait evolution, varying the parameter configuration. Because we estimated that most transitions would occur from the HIV-B consensus amino acids to non-HIV-B consensus amino acids, we investigated if a unidirectional model, which did not allow the reverse transition, would fit better than a bi-directional model. Because we believe that these four subtype-specific positions are undergoing roughly the same process, we specified a hierarchical prior on their transition rates. Implementing the hierarchical prior was trivial in the unidirectional case, as there we could set the B→non-B transition rates to 1, and the non-B→B transition rates to 0, and put a hierarchical prior on the clock rates. The bi-directional models were parameterized by setting the B→non-B transitions to 1 and allowing the non-B→B transitions to vary as a relative rate; this model was made hierarchical by allowing the clocks to vary freely and independently, while hierarchical priors were placed on the relative rate for each subtype-specific site. We used the stepping stone sampling procedure implemented in BEAST to obtain estimates of the marginal likelihood for each of the four models, which were converted to Bayes Factors for model comparison by taking the difference between the marginal likelihoods of two models on the log scale (Baele et al. 2012, 2013). XML files are available upon request.

### 4.18 Calculating the fraction of African Americans in the US HIV-infected population over time

To estimate the ethnic demographics of the HIV-infected population in the USA, we converted existing estimates of the ethnic demographics of HIV incidence (new infections) to prevalence (number of living hosts infected). We obtained incidence data from the literature (Hall et al. 2008; Prejean et al. 2011) and used the following assumptions:

1. In the beginning of the epidemic (when no drugs were available) the average survival time was 10 years.
2. Starting from 1996 the availability of combination therapy increased the survival time to at least 25 years.
3. Starting from 2002 further improvement in the standard of care extended this to at least 32 years.

These assumptions were the same for all ethnic groups.

We then calculated the fraction of African Americans in the US HIV-1 infected population at each time point.

### 4.19 Regression of the US HIV-Infected population’s African American frequencies on time

To smooth the data and to incorporate uncertainty in the estimates, we performed a Bayesian regression of time on the estimated African American proportions of HIV-1 infected Americans (Supplementary Fig. S11). Initial observations revealed a change (or a change-point) between growth and decay of the proportion of African Americans around 1998 and we used a Bayesian change-point analysis to model this. To prevent discontinuity at the change-point, it was required that the function was continuous with a continuous first derivative, and we, therefore, chose the following form:

$$f(x)=\alpha_{0}+\alpha_{1}(x-p)+\alpha_{2}(x-p{)}^{2}+\alpha_{3}(x-p{)}^{3}+\alpha_{4}(x-p{)}^{4}$$

 

$$g\left(x\right)=c+de^{\gamma \left(x-p\right)}$$

 

$$y=\left\{\begin{array}{cc} f(x) & if\;x\leq p\\ g(x) & \mathrm{otherwise}. \end{array}\right.$$

where:

$$\alpha_{0}=c+d$$

 

$$\alpha_{1}=d\gamma$$

We used normal priors of mean 0 and standard deviation 10 on all parameters, except the *p* change-point parameter (normal with mean corresponding to the year 1998 and SD 1), because the approximate position of the change-point is clearly between 1996 and 2000, and the likelihood function precision parameter (gamma with shape = 0.001 and rate = 0.001). To stabilize the variance and thereby ensure homoscedasticity, a regression model was fitted, which was weighted by the total number of patients alive according to the simple population model; this had the further advantage of allowing some uncertainty around the earlier time points. The calculated frequency of African Americans in the US HIV-infected population was standardized, and 1980 was taken as Year 0. Because the model uses latent binary variables to distinguish between the *f* and *g* functions we used the JAGS Gibbs sampler (Plummer 2003). Because of strong correlations between the parameters (due to collinearity of the (*x*–*p*) polynomials), it was necessary to run JAGS for 100,000 steps. The median fitted values from this regression were used as the explanatory variable in the subsequent Bayesian Multi-level Model.

### 4.20 Bayesian multi-level model incorporating phylogenetic information

We included a phylogenetic signal in much the same way as in the previous analysis, by fitting parameters for every position/patient combination. These parameters were sampled from a single multivariate normal distribution for every subtype-specific position, using the phylogenetic correlation matrix to relate patients according to the evolutionary history of the virus. This can be expressed as follows:

$$y_{ij}∼\mathrm{Bernoulli}(p_{ij})$$

 

$$\mathrm{logit}(p_{ij})=\alpha_{j}+\beta_{j}x_{i}+u_{z_{i}j}$$

The (hyper-)priors are:

$$\alpha_{j}∼N(0,2)$$

 

$$\beta_{j}∼N(\lambda ,\sigma )$$

 

λ∼N(0,2)

 

σ∼Half-Normal(0,1)

 

$$u_{*j}∼N_{m}\left(0,C\right)$$

This creates a multi-level model where each data point informs both the estimated probability of HIV-B-likeness for a subtype-specific position in an individual patient (through the $u_{ij}$ parameters) and the population level estimates (through $\beta_{j}x_{i}$). Additionally, we used a hierarchical prior on the slopes to relate the four subtype-specific sites to one another, creating what would be in classical statistical terms a random slope model, which allows the partial pooling of information to further protect against phylogenetic covariance as well as giving us additional power. This construction is justified by our prior belief that these four positions are special in the same way, and by our explorations of the data set when testing the phylogenetic model, which favoured a hierarchical prior on the transition rates. A hierarchical prior on the transition rates is the phylogenetic analogue to the hierarchical prior on the regression coefficients implemented here. More generally, a hierarchical prior can also be seen as a conservative choice due to the protection it confers against multiple hypothesis testing (Gelman 2009). As in the previous worldwide analyses, we used the correlation matrix as a variance–covariance matrix to prevent non-identifiability, implicitly setting the σ in σC to 1 (not shown in Equation (4) to avoid confusion with the β variance σ). The model was fitted using Stan and ran for 5,000 steps using a non-centred parameterization and the MCMC chains converged on the posterior distributions (all $\hat{R}$≤1.01). To validate the generative properties of the model, we performed a posterior predictive test, which confirmed that the actual data could have been generated by our model. Due to the binary nature of our data, we took the proportion of HIV-B consensus amino acids in each position for every patient in each year and calculated the yearly average for each subtype-specific position separately.

### 4.21 Population-level estimates of HIV-B consensus amino acid frequencies

Using our multilevel model, we reconstructed the population-level proportion estimates of the HIV-B consensus amino acid over time. We set *x'* as the inferred African American frequency in every year between 1982 and 2009 and used the following formula, which only included the population level parameters in the multilevel model and left out the patient-specific adjustments:

(6) $$\mathrm{logit}(q_{ij})=\alpha_{j}+\beta_{j}x{'}_{i}$$

By plotting these population-level estimates ($q_{ij}$) against time, we recovered the underlying pattern from the data, which was the population level estimate that can be visualized together with the uncertainty inherent in the estimate (Fig. 5).

## 5. Data sets and code availability

The data were obtained from publicly available databases (the LANL HIV sequence database (Foley et al. 2018) and the Allele Frequency Net database (Gonzalez-Galarza et al. 2015)), and the South African Zulu HLA data were kindly shared by Professor Bruce Walker, Harvard University, and Professor Mary Carrington, Frederick National Laboratory for Cancer Research. All new code and algorithms are available upon request .

## Supplementary data

Supplementary data are available at *Virus Evolution* online.

## Author contributions

A. K. N. I. conceived and designed the overall study. N. C. K. and P. L. conducted the phylodynamic analyses. N. C. K., D. L., B. L, S. C., and A. K. N. I. performed data analyses and modelling and A. K. and P. L. contributed intellectual input. A. K. N. I. and N. C. K. wrote the paper; all authors commented on and approved the final article.

## Supplementary Material

veaa085_Supplementary_Data
