# Supplementary material for: HIV-1 p24Gag adaptation to modern and archaic HLA-allele frequency differences in ethnic groups contributes to viral subtype diversification
Source: Virus Evol. 2020 Dec 12;6(2):veaa085. doi: 10.1093/ve/veaa085 (PMC7733611; doi:10.1093/ve/veaa085)
Supplement: veaa085_Supplementary_Data [file veaa085_supplementary_data.pdf]

Supplementary data:

HIV-1 p24Gag adaptation to modern and archaic HLA-allele frequency differences in ethnic groups contributes to viral subtype diversification

Nicolaas C. Kist, Ben Lambert, Samuel Campbell, Aris Katzourakis, Daniel Lunn,
Philippe Lemey, Astrid K.N. Iversen

Overview:

1) Supporting figures 1 to 12:

Fig. S1: Subtype-specific differences between the two conserved, clinically important HIV Gag Regions in HIV-B (B) and HIV-C (C)

Fig. S2: Outline of HLA-associated single-epitope and multiple-epitope selective pressures.

Fig. S3: HIV-1 subtype distribution in Sub-Saharan Africa

Fig. S4: The patients' haplotype score in PC1 and PC2

Fig. S5: HIV-C, HIV-B and selected HLA frequencies in Southern Africa, the US, and Europe

Fig. S6: HIV-B phylogeny

Fig. S7: HIV-C phylogeny

Fig. S8: MCA of each patient's HIV-1 subtype-specific amino acids

Fig. S9: Modeling of the evolution of HIV-C SSAA using HIV-B-related HLA ORs

Fig. S10: HLA diversity in mixed African American and US Caucasian populations

Fig. S11: Proportion of African Americans in the US HIV-infected population over time

Fig. S12: Phylogeny of the American HIV-B epidemic combined with demographic data

2) Supporting tables 1 to 8:

Table S1: African populations with HLA A, B, and C data

Table S2: The number of p24 Gag sequences per country and the number of HIV-1 subtypes, CRFs and URFs per country and the country code abbreviation key

Table S3: Complete key to the African ethnicities in **Fig. 3C**

Table S4: Complete key over HIV-1 subtypes shown in **Fig. 3D**

Table S5: Complete key over African languages shown in **Fig. 3E**

Table S6: HLA class I allele frequencies in worldwide populations

Table S7: Overview of the amino acid variation at SSP in HIV-1 subtype consensus sequences

Table S8: LANL patient IDs of patients with imputed four digit HLAs

Figure S1

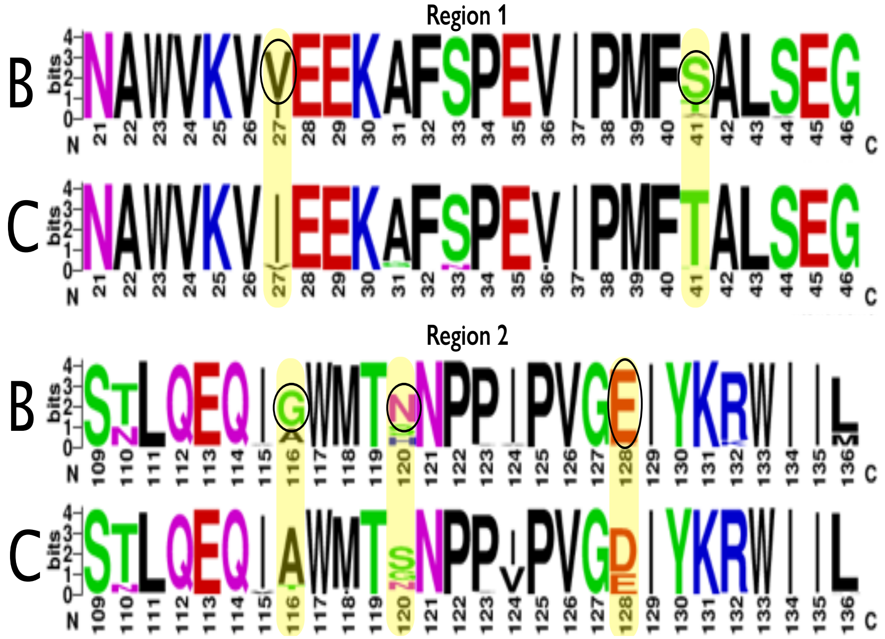

Fig. S1: Subtype-specific differences between the two conserved, clinically-important HIV Gag Regions in HIV-B (B) and HIV-C (C)

The subtype-specific positions (SSP) 27, 41, 116, 120, and 128 are highlighted in yellow, and the letters representing site-specific amino acids are sized according to their associated frequencies. HIV-B position 128 and HIV-C positions 27, 41, and 116 are almost always occupied by the consensus amino acids and cannot be used in the analysis. The HIV-B consensus amino acids are circled. This figure was generated using a single sequence from each HLA annotated patient in the HIV database (Foley et al. 2018).

Figure S2

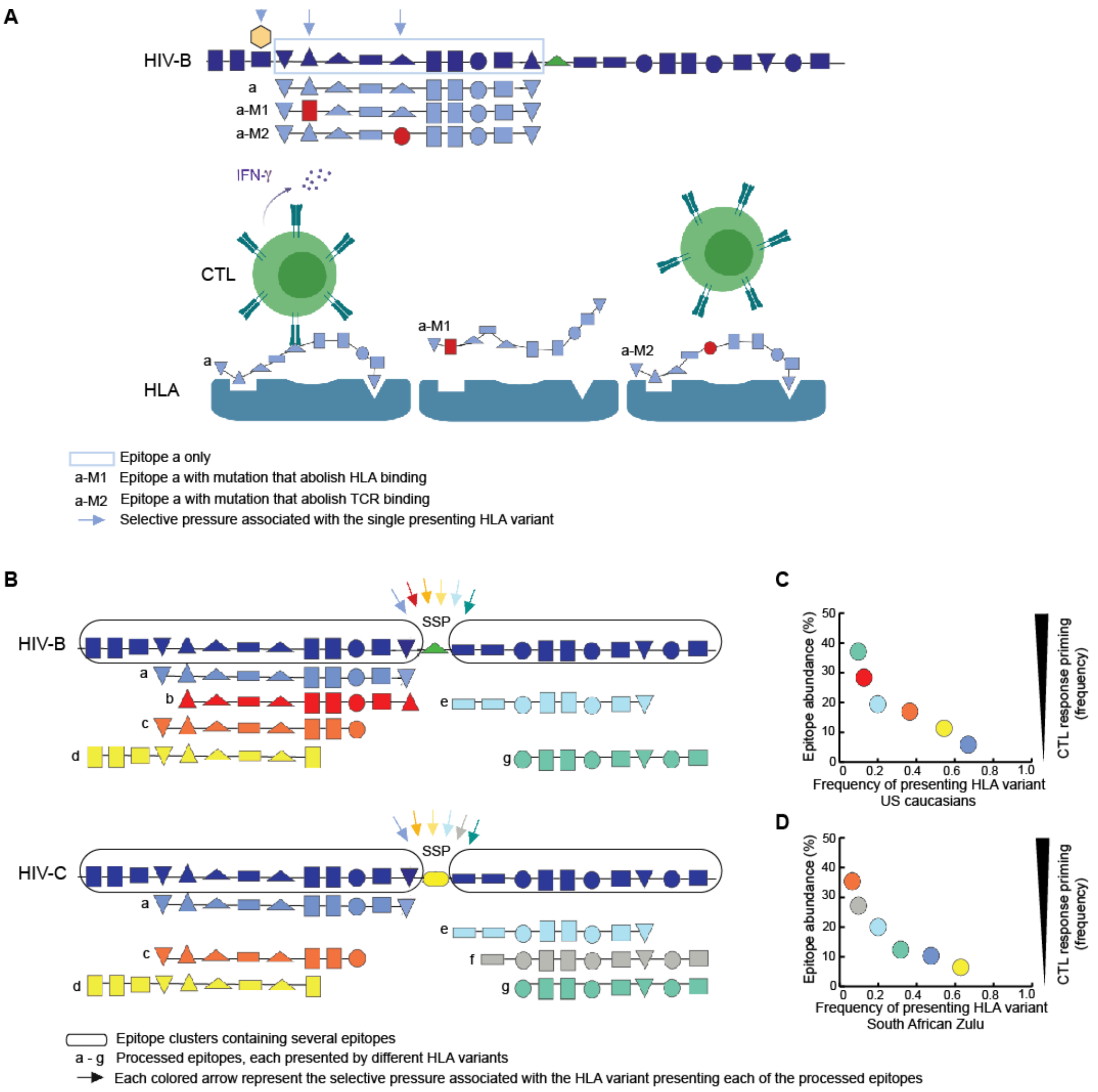

Fig. S2. Outline of HLA-associated single-epitope and multiple-epitope selective pressures. **(A).** Three common forms of HLA-associated single epitope selective pressures. CTL responses targeting an epitope might select for an upstream mutation (indicated by the orange polygon) that disrupts ERAP trimming (as in (Draenert et al. 2004)) or intra-epitope mutations that abrogates epitope binding to the presenting HLA molecule or T cell receptor (TCR) recognition of the epitope (reviewed in (Goulder and Watkins 2008; Goulder and Walker 2012)). Arrows signify different forms of CTL selective pressures, geometric symbols indicate amino acids, and the green triangle represents an HIV-B subtype-specific amino acid (as in (**B**)).

(B). Schematic representation of a conserved p24Gag region in HIV-B and HIV-C with a subtype-specific amino acid position (SSP; HIV-B, green triangle, HIV-C, yellow ellipse) and down- and upstream epitope clusters; epitopes a-g indicate epitopes processed by intra-cellular proteasomes. When a patient presents any of these epitopes, additional HLA-associated selective pressures might select for the intra-epitope CTL-escape mutations described in **Figure S2A**. The nature of the amino acid in the subtype-specific position controls intra-cellular proteasomal production of the surrounding epitope-clusters (Tenzer et al. 2014), which each can contain 20-50 epitopes presented by approximately as many HLA variants (Foley et al. 2018). The outlined structure consisting of partly overlapping epitope sequences in clusters in hydrophobic regions that are separated by a subtype-specific position is also common in other HIV-1 proteins (Foley et al. 2018; Lucchiari-Hartz et al. 2003).

(C), (D). Schematic outline of the outcome of the HLA-associated selective pressure on the subtype-specific positions (experimentally demonstrated in (Tenzer et al. 2014)). The combined HLA-associated selective pressure on the SSP in an HIV-infected population results in an inverse relationship between the abundance of a processed epitope and the frequency of the presenting HLA allele in the population in which the virus circulates (Tenzer et al. 2014). In this hypothetical example, the grey epitope is not produced when the proteasome processes HIV-B because the HLA variants that can present the grey epitope is very common in US Caucasians. In contrast, the red epitope is not produced when the proteasome digests HIV-C because the restricting HLA variant is very common in the South African Zulu population. The likelihood of CTL priming will increase with the amount of presented epitope on the infected cell surface (Faroudi et al. 2003; Tenzer et al. 2009).

Figure S3

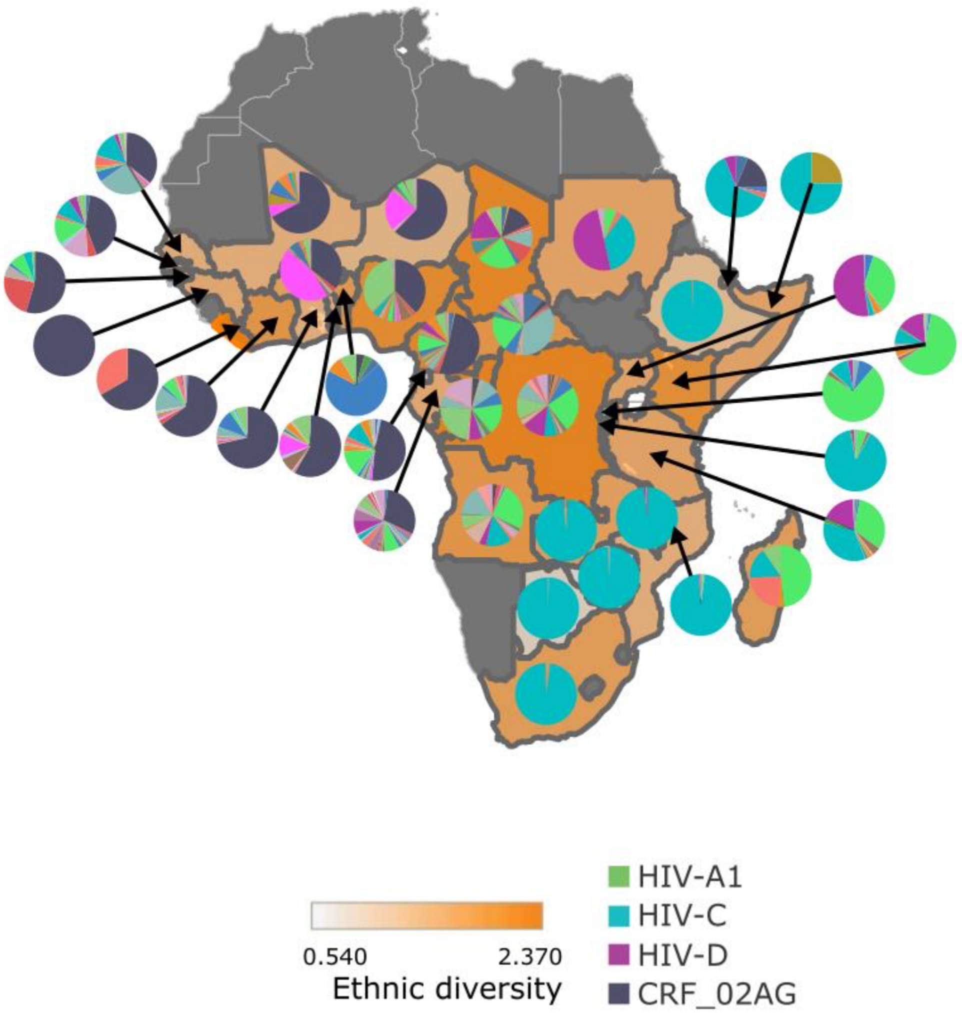

Fig. S3: HIV-1 subtype and CRF distribution in Sub-Saharan Africa

This figure is similar to **Fig. 3d**, but the pie charts have not been scaled according to the number of sequences from each country. The map showing the ethnic diversity within Sub-Saharan Africa (orange shaded background) is overlaid with pie charts demonstrating HIV-1 subtype diversity within each country. Missing countries (dark grey) lacked either HIV-1 or ethnic fractionalization data; complete subtype key can be found in **Table S4**.

Figure S4

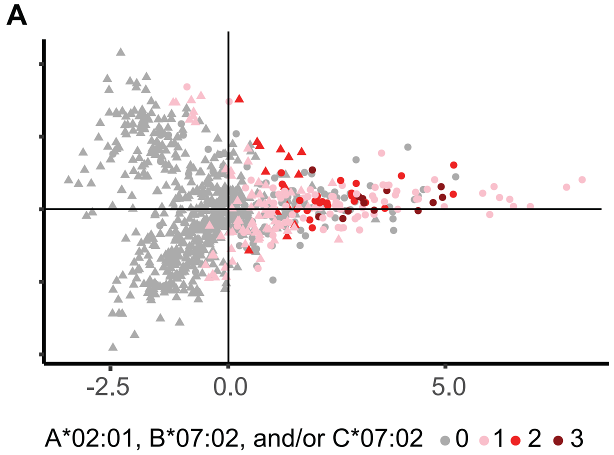

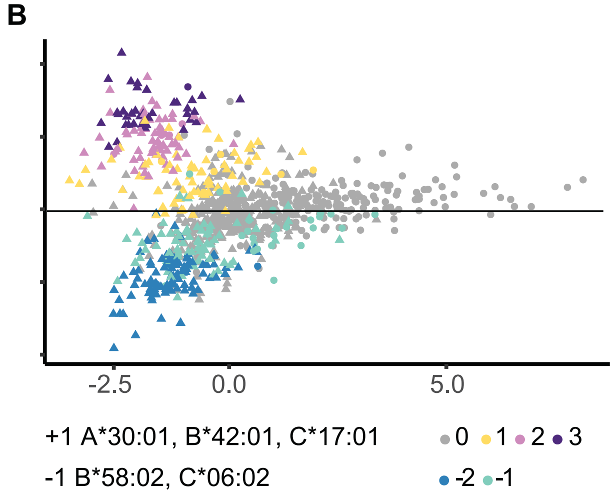

Fig. S4: The patients' haplotype score in PC1 and PC2

(A). The first two PCs explained 14.5% of the variance (PC1 = 7.7%, PC2 = 6.8%). The haplotype score is not stratified for Caucasian HLA variants as the HLA A*02:01 contributes most to the variance. HLA B*07:01 and HLA C*07:01 are in LD in Caucasians (a haplotype inherited from Neanderthals (Abi-Rached et al. 2011)), but not in Africans (Gonzalez-Galarza et al. 2015).

(B). The patient's haplotype score is incremented by one for each allele from haplotype HLA A*30:01-B*42:01-C*17:01 and reduced by one for each allele from haplotype HLA B*58:02-C*06:02 showing near-complete stratification of PC2 due to these haplotypes.

Figure S5

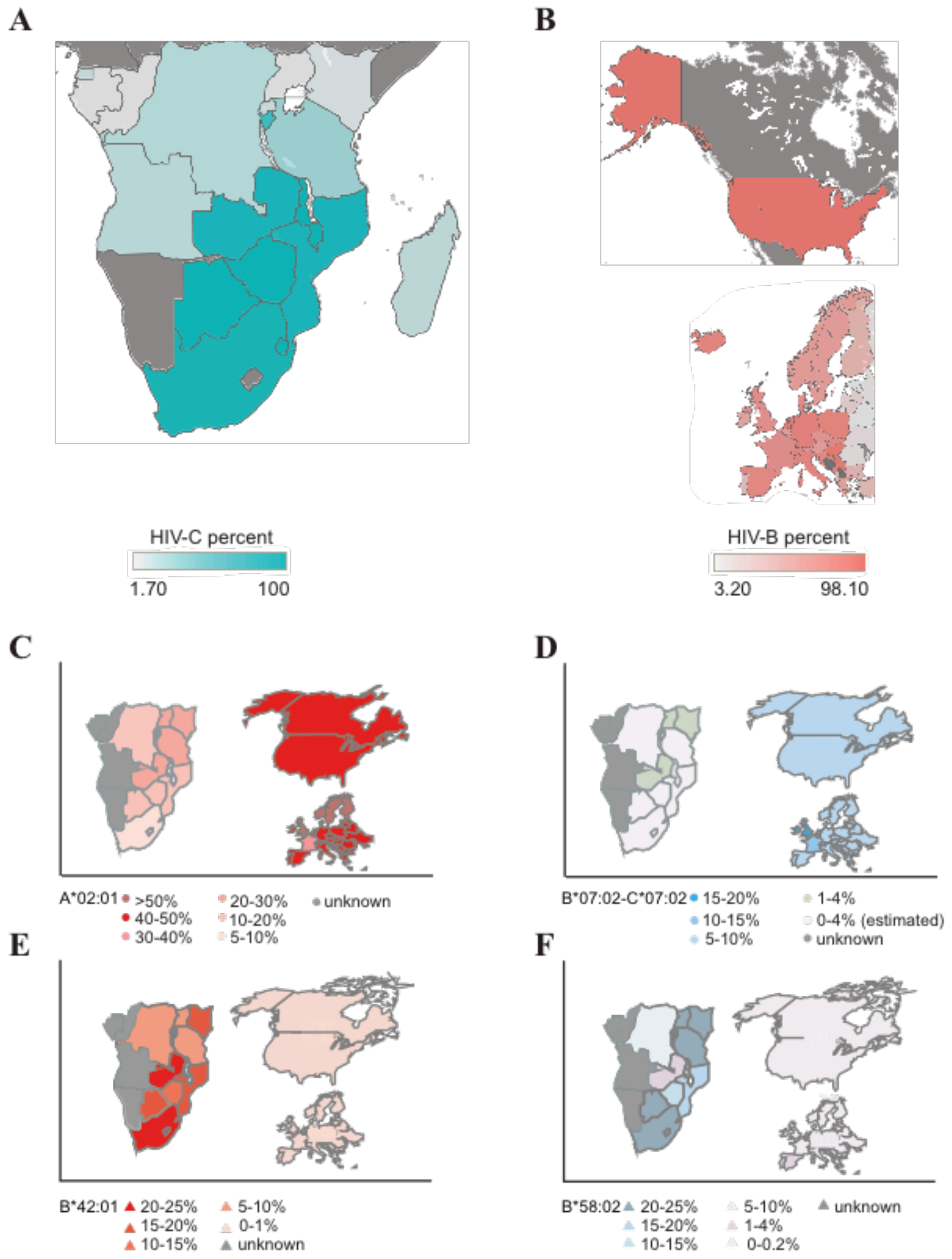

Fig. S5: HIV-C, HIV-B and selected HLA frequencies in Southern Africa, the US, and Europe

(A). The percentage of HIV-C in countries in Southern African. Dark grey signifies either no data or that the region is outside the geographical region of interest.

(B). The percentage of HIV-B in the US and Europe. The percentage of non-HIV-B sequences in Europe is higher than in the US due to a higher proportion of non-B HIV-1-infected immigrants and refugees primarily from Africa. Furthermore, the epidemic in some European countries was in part, or mostly, founded by non-B subtypes; for example, the percentage of HIV-B is low in Russia and former Soviet Union countries due to the introduction of HIV-A from the DRC and HIV-G in Portugal because of the introduction of HIV-G from Cape Verde, a former Portuguese colony (Beloukas et al. 2016; Diez-Fuertes et al. 2015).

(C-F). The HLA frequencies of key HLA variants in PC1 and PC2. Note the low frequency of HLA-B*58:02 in Zambia where the epidemic has lasted longer than in South Africa, and where HLA B*42:01, but not HLA B*58:02, is associated with facilitated HIV-1 transmission. The HLA B*07:02-C*07:02 haplotype was acquired from Neanderthals and is found primarily in Eurasians (Abi-Rached et al. 2011). HLA B*07:02 and HLA C*07:02 can be found individually in other combinations in some African ethnic groups (Gonzalez-Galarza et al. 2015).

Figure S6

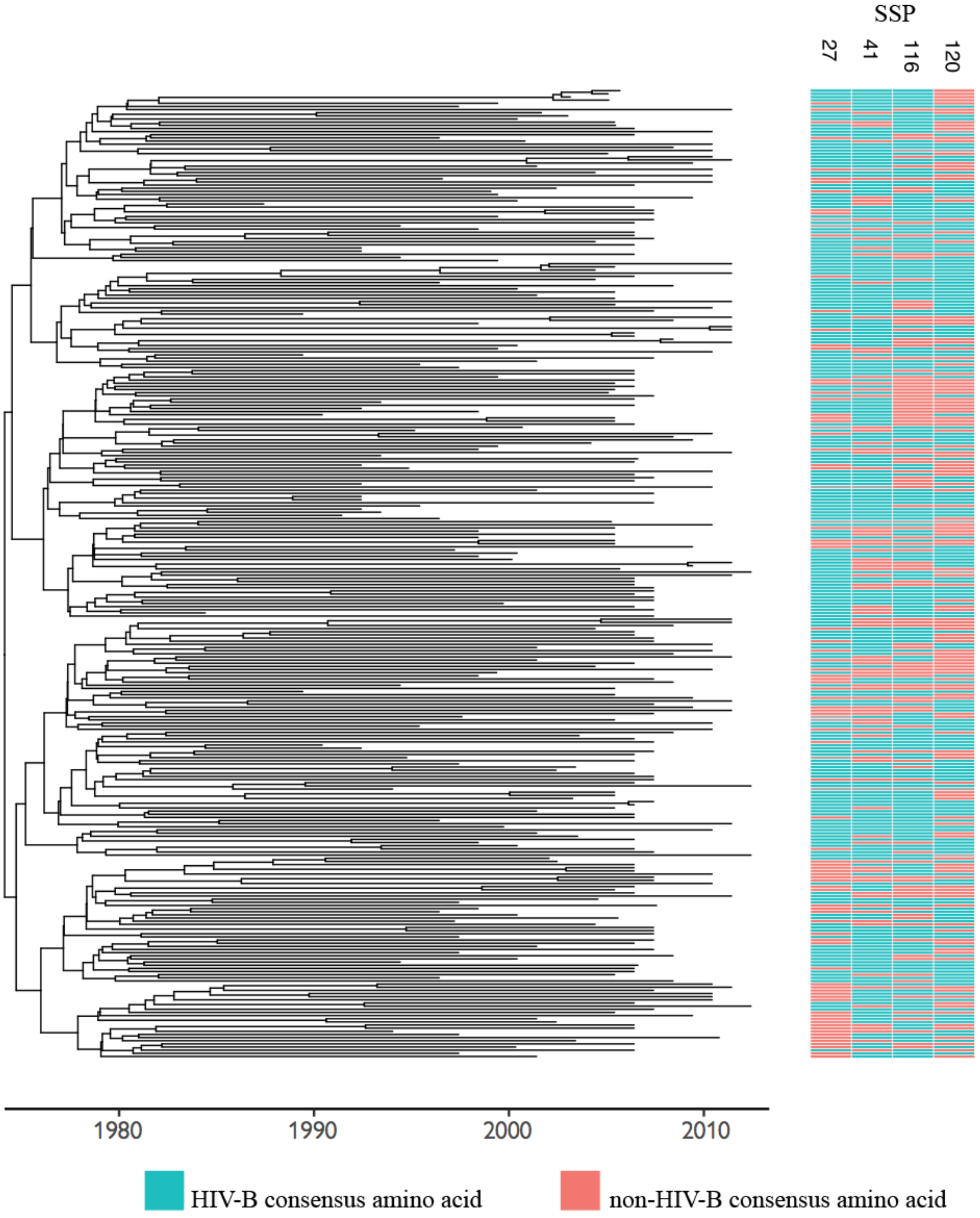

Fig. S6: HIV-B phylogeny

The HIV-B BEAST Maximum Clade Credibility phylogeny annotated with the subtype-specific positions (SSP) 27, 41, 116, and 120 (see **Fig. S1**). Every leaf-node on the phylogeny represents a patient, and the four lines next to the leaf node show whether the majority rule amino acid in each

of the four eligible SSPs was identical to the HIV-B consensus amino acid. The amino acid patterns shown here follow the phylogeny (i.e., continuous patches of red are more vertical than horizontal, and are therefore shared between closely related sequences rather than shared between multiple positions on the same patient), and demonstrate the necessity of using a multiple response random effect model where each amino acid is allowed to evolve independently. If the color pattern had been more horizontal, that would have meant a single patient's subtype-specific amino acids tended to change as a block, in which case a single patient parameter (random effect model) would have been more appropriate than the multiple random effect phylogenetic model used in this study.

Figure S7

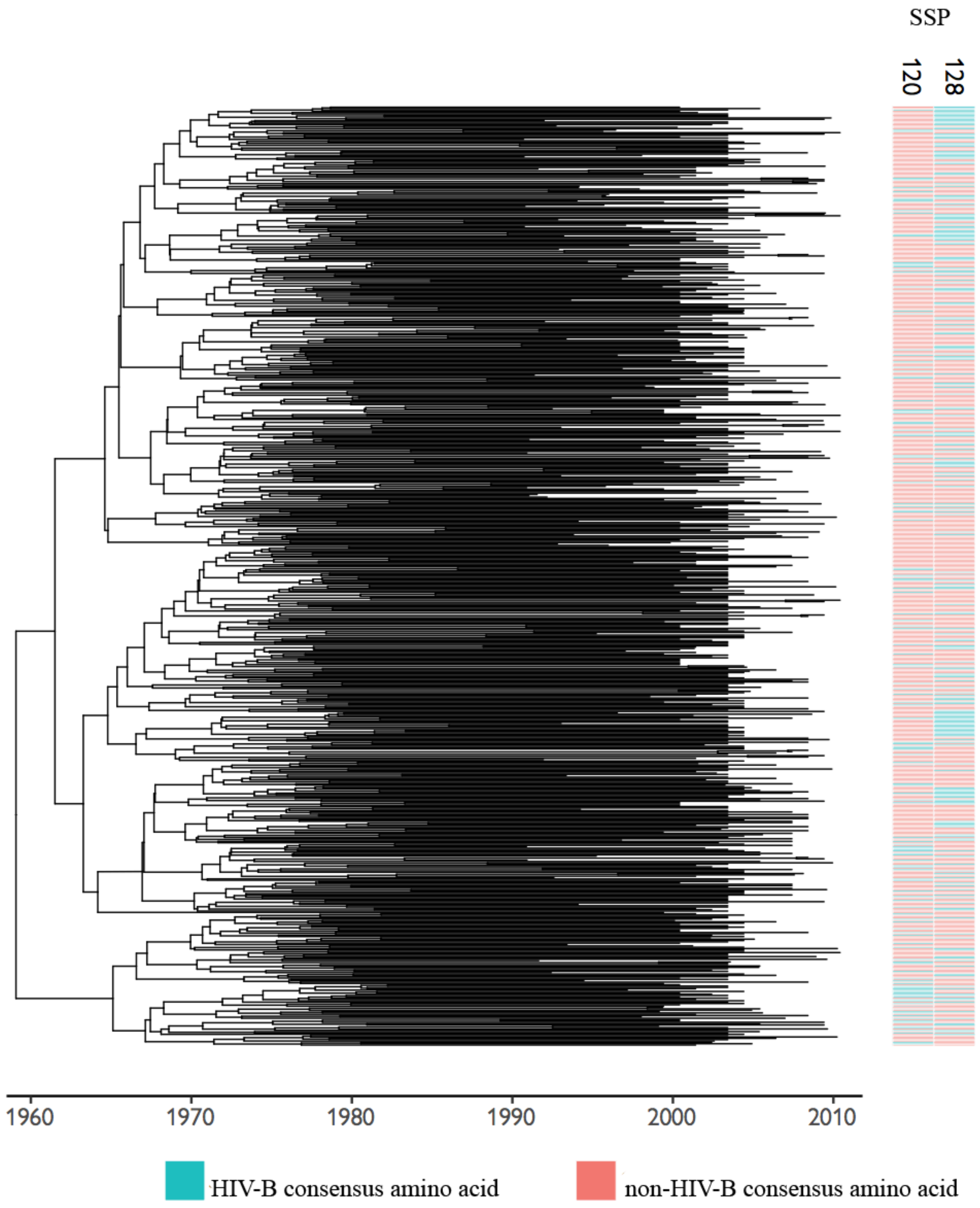

Fig. S7: HIV-C phylogeny

The HIV-C BEAST Maximum Clade Credibility phylogeny annotated with the subtype-specific positions (SSP) 120 and 128. Patients, where the majority rule amino acid is the same as the HIV-B (see fig. S1), are colored blue; all other amino acids are colored red.

Figure S8

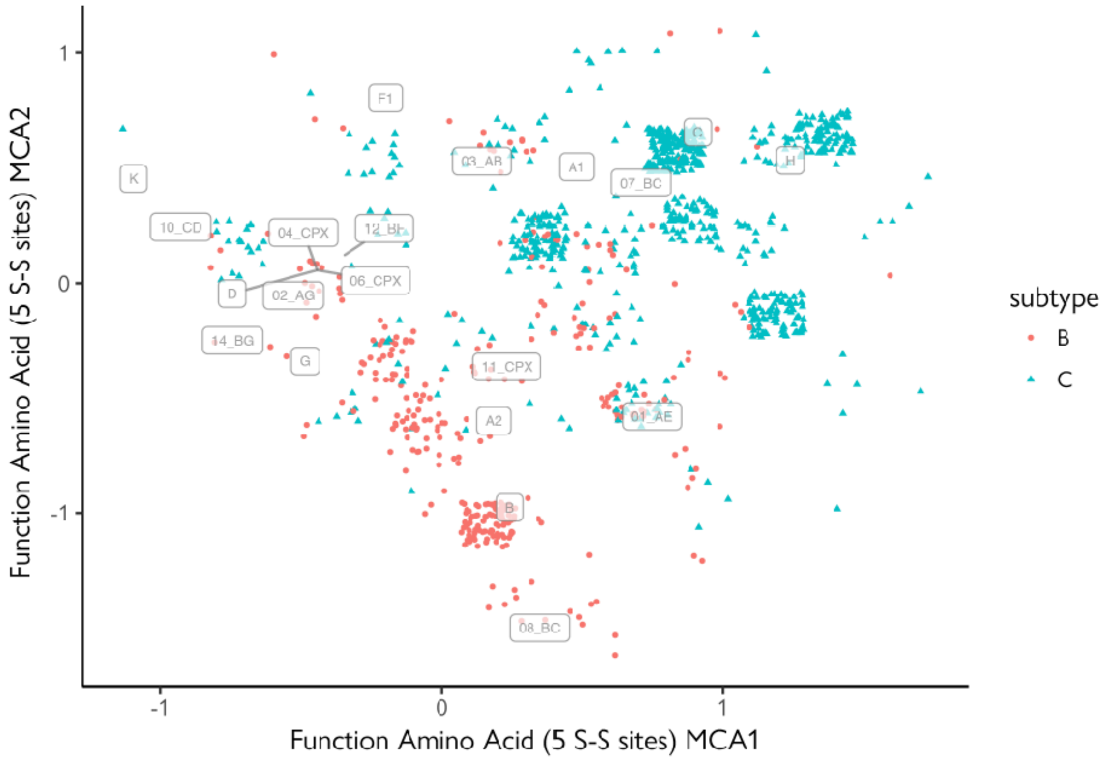

Fig. S8: MCA of each patient's HIV-1 subtype-specific amino acids

The MCA converts each patient's HIV subtype-specific position amino acid profile into two dimensions. This MCA was trained using the subtype consensus sequences (labels), the MCA was then used to project the patient's HIV subtype-specific amino acids onto the two-dimensional space. Points were jittered to prevent them obscuring one another. While many patients' subtype-specific amino acids are identical to the subtype consensus (see large clusters around the HIV-B and HIV-C labeled consensus sequences), other HIV-B and HIV-C sequences are identical in the five Gag subtype-specific sites studied here (e.g., around the labeled HIV-1 circulating recombinant (CRF) 01_AE consensus).

Figure S9

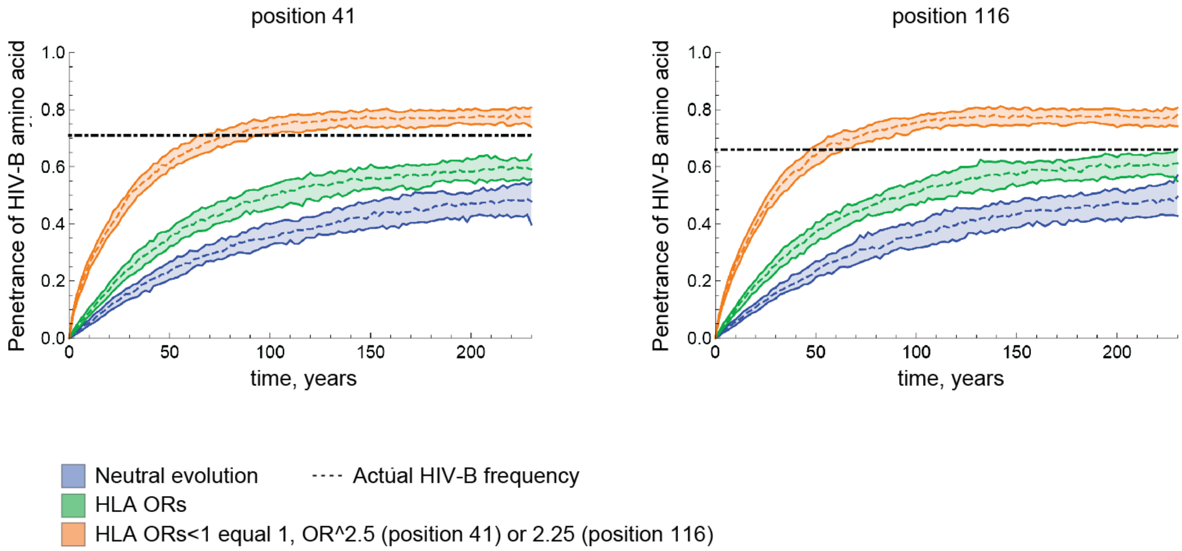

Fig. S9: Modeling of the evolution of HIV-C SSAA using HIV-B-related HLA ORs

We used an agent-based model (**1.3**) to estimate the effect of neutral and HLA-mediated selection pressures, respectively, on the evolution of p24Gag positions 41, and 116 on a fictive HIV-1 with HIV-C-like subtype-specific amino acids (SSAAs). For position 41, raising the odds to 2.9 or 3.0 made little difference. These results suggest that other, unidentified, HLA variants might influence the evolution of these positions and/or that structural co-evolutionary factors might be in play.

Figure S10

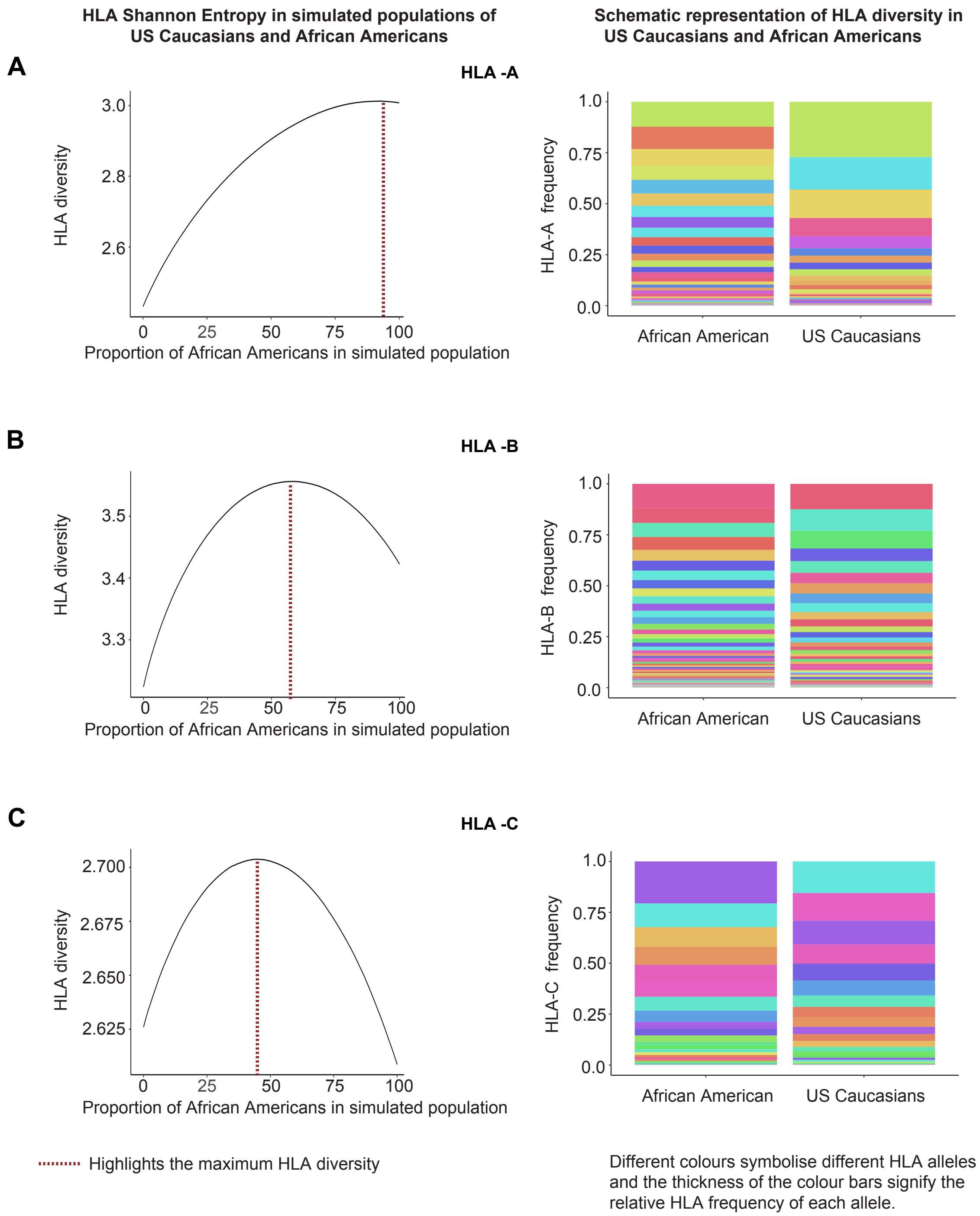

Fig. S10: Simulated mixtures of African Americans and US Caucasians (left) and HLA diversity in African American and US Caucasian populations (right)

(A). HLA A diversity in simulated populations with different proportions of African Americans and US Caucasians, and the HLA A variant frequencies in African Americans and US Caucasians (modified from (Gragert et al. 2013)).

(B, C). As in **A** for **B** (HLA B), and **C** (HLA C).

Figure S11

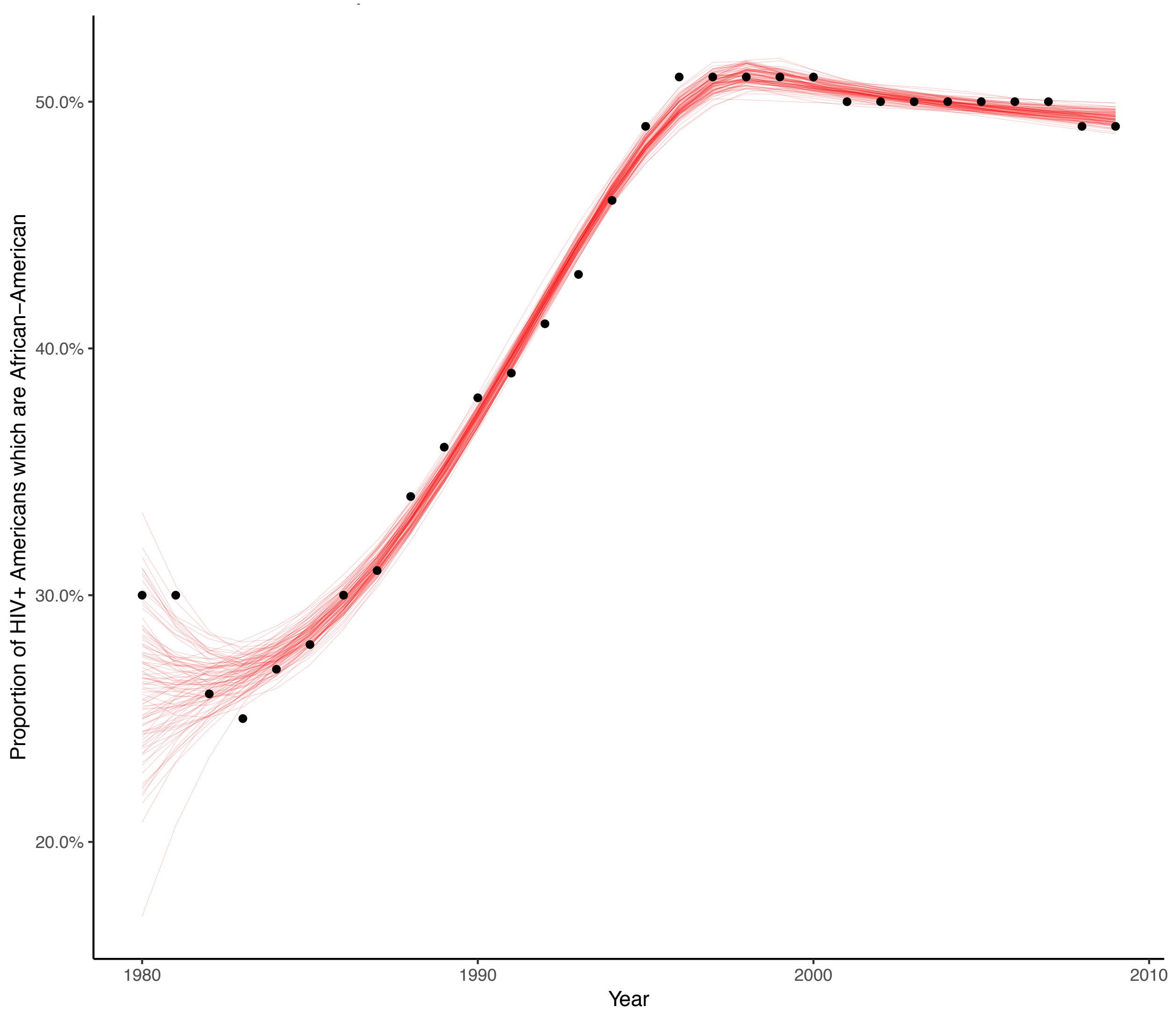

Fig. S11: **Proportion of African Americans in the US HIV-infected population over time**

The ethnic demographic proportion of African Americans in the US HIV-infected population (data from (Hall et al. 2008) and (Prejean et al. 2011)) as calculated in the simple population model with the proportion of African Americans shown as points. Samples drawn from the posterior distribution of the change-point model are shown as lines. Note the increased uncertainty around 1980 due to data limitations.

Figure S12

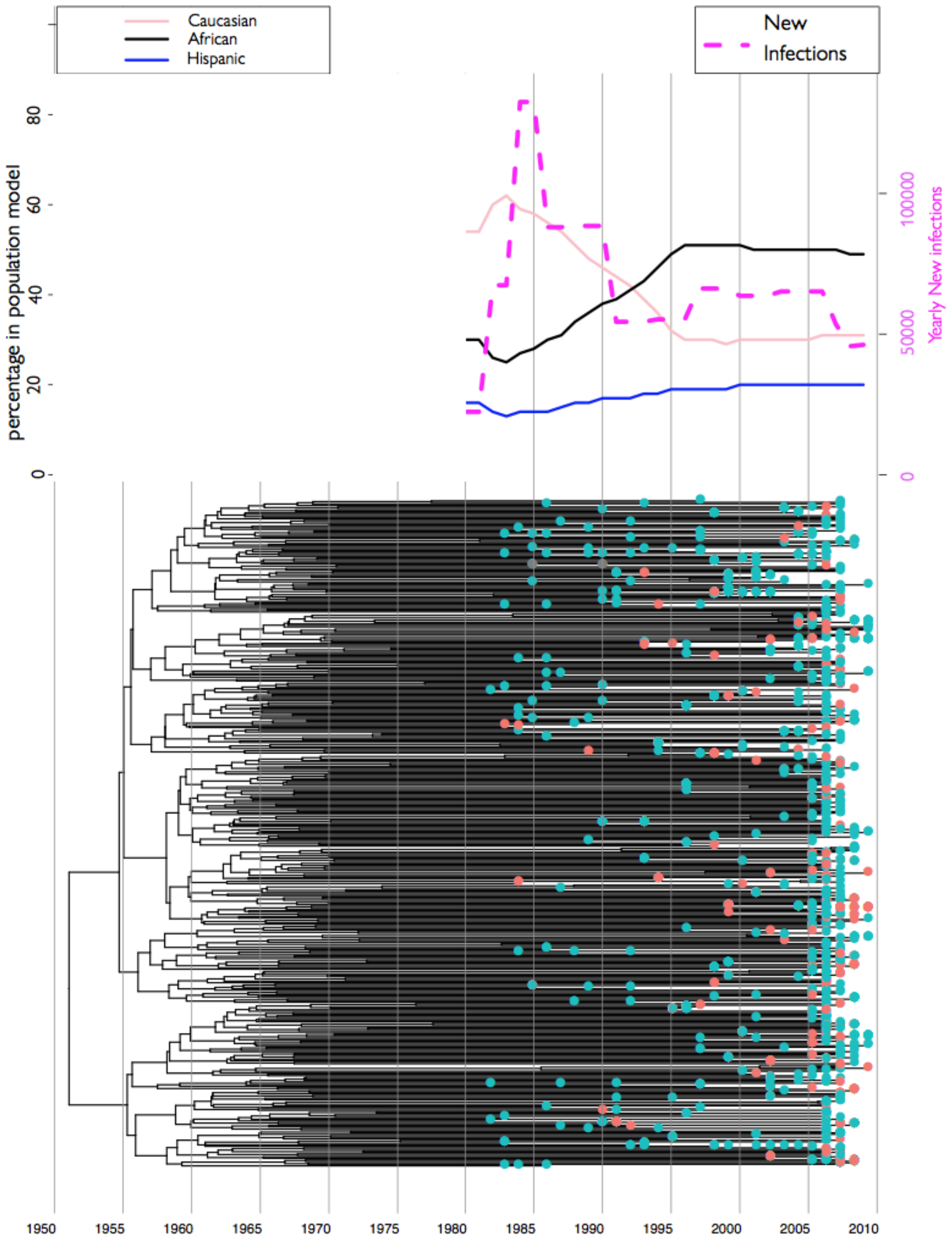

Fig. S12: Phylogeny of the American HIV-B epidemic combined with demographic data

The Maximum Clade Credibility tree, annotated with individual sequence data for position 27 (bottom, blue if a sequence is identical to the HIV-B consensus amino acid; red otherwise). In this case all non-B consensus amino acids were similar to the amino acid found in the HIV-C

consensus sequence due to the limited variation possible at this position (**Table S8**). The phylogeny was generated using one sequence per patient, but position 27 amino acid data from multiple sequences sampled from the same patient have been superimposed onto the phylogeny. These annotations illustrate the gradual increase of non-HIV-B consensus amino acids on position 27 as the African-American proportion of HIV-infected individuals in the United States increases (black line, top figure). No demographic data are available before 1980.

Table S1: African populations with HLA A, B, and C data

Table showing the only African populations with publicly available 'gold standard' HLA data (i.e., all HLA types were reported as 4-digits HLA variants)(Gonzalez-Galarza et al. 2015).

Number	Population or ethno-linguistic group	Country	Number of people in cohort
1	Bandiagara	Mali	138
2	Ga-Adangbe	Ghana	131
3	Kenya* (likely Masai)	Kenya	144
4	Luo	Kenya	265
5	Nandi	Kenya	240
6	South Africa Black**	South Africa (Free State)	200
7	Zulu	South Africa (KwaZulu Natal)	606
8	Kampala (pop 2)***	Uganda	175

* The population named 'Kenya' has no ethnic group specifications but the location coordinates (obtained from the database (Gonzalez-Galarza et al. 2015)) are situated within the Masai ethnic group's territory, and the population is described as 'rural.' These coordinates differ from those of the Luo and Nandi ethnic groups, and for this reason, we assumed all three groups were distinct from each other. The data have been presented in a workshop but have not been published. The Luo people in western Kenya are closely related to Luo people in Northern Uganda, and Northern Tanzania, and are part of a larger group of ethno-linguistically-related Luo people in South Sudan and South-western Ethiopia. Their language (Dholuo) belongs to the Western Nilotic branch of the Nilo-Saharan language family. The Nandi people live in Nandi Country (previously Rift Valley Province of Kenya) and speak Nandi, which belongs to the Southern Nilotic language group. The Masai people live in Northern, Central, and Southern Kenya and in Northern Tanzania. Their language (Maasai or Maa) belongs to the Eastern Nilotic language group.

** The location coordinates of the South Africa Black HLA data is situated within the Free State province of South Africa, a region where most people speak Sotho and belong to the Sotho-

Tswana people, however, in the paper by Paximadis, M et al., the HLA data is reported to represent “a cross-section of the black and Caucasian subgroups of the South African population (Paximadis et al. 2012).” The South Africa Black and Caucasian HLA data are recorded separately in the database. The number of South Africa Black ethnic groups is ~10, but the majority of the Zulu population lives in the KwaZulu Natal and Gauteng provinces (http://www.sahistory.org.za/).

*** The HLA data from Uganda does not belong to a distinct population; “All the individuals were Black Africans from Kampala, but no other ethnic affiliation was recorded” (from (Kijak et al. 2009)).”

Table S2: The number of p24 Gag sequences per country and the number of HIV-1 subtypes, CRFs and URFs per country and the country code abbreviation key

ISO alpha-2 two-letter Country code	Country name	p24 sequences / country
**BW**	Botswana	51
**CD**	Democratic Republic of the Congo	22
**CI**	Ivory Coast	6
**CM**	Cameroon	318
**ET**	Ethiopia	35
**GA**	Gabon	6
**GH**	Ghana	13
**KE**	Kenya	633
**NE**	Niger	72
**NG**	Nigeria	19
**SN**	Senegal	27
**TD**	Chad	18
**TZ**	United Republic of Tanzania	38
**ZA**	South Africa	935
**ZM**	Zambia	12

ISO alpha-2 two-letter Country code	Country name	HIV-1 subtypes, CRFs, and URFs (group M) / country
**AO**	Angola	21
**BF**	Burkina Faso	13
**BI**	Burundi	8
**BJ**	Benin	6
**BW**	Botswana	7
**CD**	Democratic Republic of the Congo	46
**CF**	Central African Republic	30
**CG**	Congo	20
**CI**	Ivory Coast	22
**CM**	Cameroon	78
**DJ**	Djibouti	6
**ET**	Ethiopia	7
**GA**	Gabon	33
**GH**	Ghana	18
**GM**	Republic of the Gambia	10
**GN**	Guinea	1

**GQ**	Equatorial Guinea	16
**GW**	Guinea Bissau	7
**KE**	Kenya	35
**LR**	Liberia	2
**MG**	Madagascar	5
**ML**	Mali	10
**MW**	Malawi	8
**MZ**	Mozambique	8
**NE**	Niger	13
**NG**	Nigeria	23
**RW**	Rwanda	10
**SD**	Sudan	5
**SN**	Senegal	35
**SO**	Somalia	2
**TD**	Chad	19
**TG**	Togo	21
**TZ**	United Republic of Tanzania	19
**UG**	Uganda	33
**ZA**	South Africa	30
**ZM**	Zambia	14
**ZW**	Zimbabwe	7

Data were obtained from the HIV data base (Foley et al. 2018).

Table S3: Complete key to the African ethnicities in Fig. 2C

- ABABDAH
- ABBALA
- ABE
- ABIDJI
- ABINSI
- ABO
- ABRON
- ACIPA
- ACOLI
- ADARAWA
- ADJUKRU
- AFAR
- AFO
- AGAR
- AGATU
- AGOI
- AGUA
- AHANTA
- AIKE
- AIT WARAIN
- AJA
- AKAN
- AKANIKI
- AKARE
- AKEBON
- AKOKO-EDO
- AKOOSE
- AKPA
- AKPA-YACHE
- AKPES
- AKPINI
- AKPOSO
- AKYE
- ALAGO
- ALEGE
- ALGERIAN
- ALUR
- AMARAR
- AMBA
- AMBO
- AMBOIM
- ANAANG
- ANFILLO
- ANGAS
- ANGERE
- ANIA
- ANO
- ANTANDROY
- ANTANUSI
- ANTEMURU
- ANTESAKA
- ANUAK
- ANUFO
- ANYI
- AOUDJILA
- AOUK
- APAKIBETI
- ARAD
- ARGOBA
- ARIAAL
- ARUM
- ARUSI
- ASANFWE
- ATEN
- ATSAM
- ATTA
- ATUOT
- AULYEHAN
- AUSHI
- AUYUKAWA
- AVIKAM
- AVOKAYA-AJIGU
- AVOKAYA-OJILA
- AWAK
- AWIYA
- AWUTO
- AYIGHA
- BA
- BAALI
- BABALE
- BABANKI
- BABILE
- BABOLE
- BADA
- BADE
- BAGA
- BAGA MANDORI
- BAGGARA
- BAGHAP
- BAGIRMI
- BAHARIYA
- BAI
- BAILO
- BAILUNDU
- BAINUK
- BAKA
- BAKO
- BAKPINKA
- BAKUNDU
- BAKWE
- BAKWELE
- BALANTA
- BALONG
- BAMBARA
- BAMILEKE
- BAMUN
- BANDA
- BANDI
- BANGANTU
- BANGBA
- BANGU-BANGU
- BANGWINJI
- BANNA
- BARA
- BARABRA
- BARAMBO
- BAREA
- BAREIN
- BARI
- BARIBA
- BARWE
- BASA
- BASAA
- BASHAR
- BASONGO-MENO
- BASSA
- BASSOSSI
- BATA
- BATAHIN
- BATI
- BAULE
- BAUSHI
- BAYGO
- BEBIL
- BEDAWI
- BEEMBE
- BEKWARRA
- BELANDA
- BELANDA VIRI
- BELLE
- BEMBA
- BEMBE
- BENA
- BENDE
- BENGA
- BENI GIL
- BENI MENASSER
- BENI MZAB
- BENI-AMER
- BERABISH
- BERANDJOKO
- BERGDAMA
- BERIBERI
- BEROM
- BERTA
- BERTI
- BESOMBO
- BETE
- BETE-BENDE
- BETI
- BETSILEO
- BETSIMISARAKA
- BEYRU
- BEZANUZANU
- BIAFADA
- BIALI
- BIDEYAT
- BIDIANKAMBA
- BILE
- BILEN
- BIMOBA
- BINDI
- BINGA
- BIRA
- BIRGIT
- BIRIFOR
- BIRKED
- BISA
- BOBANGI
- BOBILI
- BOBO
- BOBO-OULE
- BODO
- BOFI
- BOGHOM
- BOGURU
- BOKALA
- BOKIBA
- BOKKOS
- BOKO, BUSA
- BOKONGO
- BOKYI
- BOLENDO
- BOLENGE
- BOLGO
- BOLOKI
- BOLOMBOKI
- BOLON
- BOLONGO
- BOM
- BOMASA
- BOMU
- BONDEI
- BONDJO
- BONDO
- BONDONGO
- BONGI
- BONGILI
- BONGO
- BOR
- BORORO
- BOSOKU
- BOZO
- BUA
- BUBIA
- BUDAMONO
- BUDDU
- BUDJA
- BUDUKWA
- BUDUMA
- BUILSA
- BUKA
- BULAANG
- BULALA
- BULLOM
- BUMA
- BUMAJI
- BUMALI
- BUNGU
- BURA-PABIR
- BURAK
- BURJI
- BURU
- BURUN
- BURUNGE
- BUSHOONG
- BUYU
- BWAALA
- BWARI
- BWENDE
- BWILE
- BWISI
- BYEP
- CARA
- CHAAMBA
- CHAGGA
- CHAKALI
- CHAM
- CHAMBA
- CHAOUIA
- CHE
- CHISHINGA
- CHIWERE'S NGUNI
- CHOKWE
- CHOKWE, LELE
- CHOKWE, LUNDA
- CHOPI
- CIKUMA
- CINGOLO
- CIPALA
- CISANJI
- CITATA
- CIVULA
- COMO KARIM
- CWAMHI-WURI
- DAAROOD
- DABA
- DADIYA
- DAGAARI
- DAGOMBA
- DAHALO
- DAISU
- DAJU
- DAKAKARI
- DAKHLA
- DAMA
- DAN
- DAN-GEH
- DANAKIL
- DANGYO
- DARI
- DASS
- DAY
- DCIRIKU
- DEG
- DEGEMA
- DEKAKIRE
- DELIM
- DEMBO
- DENYA
- DEY
- DIA
- DIDA
- DIDINGA
- DIGO
- DIGODIA
- DII
- DIJIM
- DIKIDIKI
- DINKA
- DIO
- DIOBO
- DIZI
- DJAGADA
- DJEBALA
- DJEM
- DJENNENKE
- DJERBA
- DODOTH
- DOE
- DOGON
- DOKA
- DOKO
- DOKO-UYANGA
- DONDO
- DONGO
- DRAWA
- DUAISH
- DUALA
- DUI-MENIA
- DUKA
- DULBU
- DULI
- DUMBULE
- DURUMA
- DUUPA
- DUWAI
- DYALONKE
- DYE
- DYULA
- DZA
- DZING
- EASTERN LUBA, KUNDA
- EBANGA
- EBIRA
- EDO
- EFIK
- EFUTOP
- EGGON
- EGYPTIAN
- EJAGHAM
- EJAMAT
- EKIT
- ELEKU
- ENDO
- ENYELLE
- EOTILE
- EPEITA
- ESAN
- ESELE
- ESHIRA
- ESIMBI
- ETON
- ETULO
- EWE
- EWONDO
- FALI
- FANYAN
- FEZARA
- FEZZAN
- FIA
- FIGIG
- FIHERENA
- FILALA
- FIPA
- FODONON
- FON-GBE
- FONGE
- FONGORO
- FOODO
- FUFULDE
- FULANI
- FULANKRIABE
- FULBE
- FULILWA
- FULIRU
- FUNGWA
- FUNGWE
- FUR
- FURU
- FYAM
- FYER
- GA'ANDA
- GAAJAK
- GAAJOK
- GAALIN
- GAAM
- GADAMES
- GADE
- GAFSA
- GAGU
- GALA
- GALAMBU
- GANAGA
- GANDA
- GANJA
- GARREH-AJURAN
- GAYA
- GBAGA
- GBAGYI
- GBANZIRI
- GBARI
- GBAYA
- GBERI
- GBII
- GBINDA
- GBIRI
- GBOMA
- GEJI
- GENYA
- GERAWA
- GERI
- GHANGALA
- GHULFAN
- GIBE
- GIDAR
- GIIWO
- GIMIRO
- GIMMA
- GIMNIME
- GIMR
- GINGO
- GISU
- GIZIGA SOUTH
- GOBU
- GODIE
- GOEMAI
- GOGO
- GOKANA
- GOLA
- GOMA
- GOMANI'S NGUNI
- GONDO
- GONJA
- GOROWA
- GOUIN
- GOWA
- GREBO
- GUA
- GUDE
- GUDU
- GUENZE
- GULA
- GULA KARA OF SUDAN
- GULE
- GURAGE
- GURENSI
- GURMA
- GURMANA
- GURO
- GURUNTUM
- GWA
- GWANDARA
- GWE
- GWENO
- GWERE
- GWOMU
- GYEM
- HA
- HADENDOWA
- HADIYYA
- HAM
- HAMAMA
- HAMBA
- HAMYAN
- HANDA
- HANGAZA
- HANYA
- HARARGE OROMO
- HARARI
- HAVU
- HAWIYA
- HAYA
- HEHE
- HEMBA
- HEMBA, BANGU-BANGU
- HEMBA, BUYU
- HENGA
- HERERO
- HIMA
- HINGA
- HOLO-HOLO
- HOLOHOLO
- HOLU
- HOMBO
- HOROM
- HUBA
- HUM
- HUNDE
- HUNGANA
- HUNGO
- HUNGWORO
- HUTU
- IBAJI
- IBIBIO
- IBINO
- ICEN
- ICHEVE
- IDOMA
- IDON
- IFUMBA
- IGALA
- IGBO
- IGEDE
- IGUTA
- IJAW
- IJE
- IKA
- IKASSA
- IKENGA
- IKIZU
- IKOMA
- IKONGO
- IKPESHI
- IKULU
- ILA
- ILA-TONGA
- ILEBO
- ILWANA
- IMBANGALA
- IMOMA
- IMRAGEN
- INJOLO
- IPANGA
- IPULO
- IRANGI
- IRAQW
- IRIGWE
- ISAAQ
- ISANZU
- ISHA
- ISOKO
- ISSA
- ISUWU
- ITSEKIRI
- ITU
- IVBIOSAKON
- IWA
- IWELLEMMEDEN
- IYEMBE
- IZDRA
- IZI
- JA' ALIYYIN GAALIIN
- JAMALA
- JANJERO
- JANJI
- JARA
- JARAWA
- JARSO
- JEKAING
- JERA
- JERID

JIBU
JIDA
JIE
JIJI
JIMI
JIMINI
JIRU
JITA
JIU
JONGA
JOOLA
JOPADHOLA
JUKUN
JUKUN KONA
JUKUN WASE
JUKUN WURKUM
JUNGO
JUR
JUR MODO
JWIRA-PEPESA
KAAMBA
KABA
KABA DEMI
KABA NAA
KABA SO
KABONGA
KABRE
KABYE
KABYLE
KADARA
KADARU
KAFFA
KAGOMA
KAGORO
KAGURU
KAJAKSE
KAKO
KAKONDA
KALAMSE
KALANGA
KALIKO
KALUNDWE
KAM
KAMANGA
KAMANTAN
KAMBA
KAMBARI
KAMBATA
KAMIR
KAMKAM
KAMO
KAMUKU
KAMWE
KANAKURU
KANDAWIRE
KANDERMA
KANEMBU
KANGO
KANTANA
KANU
KANUFI
KANURI
KANYOK
KAONDE
KAONDE, SANGA
KARA
KARABORO
KARANGA
KARE
KAREKARE
KASEM
KASENA
KASONGI
KASONGO
KASONKE
KATAB
KATYA
KAWENDI
KAYAMBA
KAYLA
KEDERU
KEENGE
KEL
KEL AHAGGAR
KEL AYR ASBEN
KEL GRES
KEL IFFORA
KELE
KENGA
KENYANG
KENYI
KERA

KERARISH
KEREWE
KETE
KETE EAST
KETE LULUA
KETE NORTH
KETE SOUTH
KEYO
KHARGA
KHUMALO
KIBET
KIGA
KIKUYU
KIM
KIMBU
KIMBUNDU
KINGA
KIONG
KIPSIKIS
KIR-BALAR
KISAMA
KISSI
KITA
KIYAK
KLAOH
KO
KOBIANA
KOFFA
KOFYAR
KOHUMONO
KOKE
KOKO
KOL BIKELE
KOLBILA
KOM
KOMA
KOME
KONGO DINGA
KONJI
KONJO
KONKOMBA
KONO
KONONGO
KONSO
KONYANKE
KOONI
KORO
KOROP
KOTA
KOTE
KOTOKO
KOTOPO
KOUYA
KPELLE
KPESSI
KRAN, NGERE
KRESH
KRIM
KUANG
KUDU
KUFRA
KUKA
KUKELE
KUKU
KUKUYA
KULANGO
KULERE
KULUNG
KUMU
KUNAMA
KUNDA
KUNDU
KUNTA
KUNUZ
KUNYI
KUPTO
KURAMA
KURANKO
KURFEI
KURI
KURUMFE
KURYA
KUSAAL
KUSHI
KUSU
KUTEP
KUTIN
KUTU
KUTURMI
KUYERI
KUYU
KWAAMI

KWAFI
KWAKUM
KWAME
KWANDI
KWANGARI
KWANGWA
KWANJA
KWANKA
KWAYA
KWERE
KWESE
LAALI
LABWOR
LADO
LAGBA
LAGUAT
LAGWAN
LAKA
LALA
LALA ROBA
LALIA
LAMA
LAMBA
LAMBYA
LAMJA
LANDUMA
LANGA
LANGO
LARU
LEELAU
LEGA
LEGBO
LEKA
LELE
LEMBWE
LEMORO
LENDU
LENGI
LENGOLA
LENJE
LESE
LIA
LIBOLO
LIGBI
LIGI
LIJILI
LIKO
LIKWALA
LIMA
LIMO
LO
LOBI
LOBI LORHON
LOGO
LOGORIK
LOI
LOKALO
LOKELE
LOKO
LOKORO
LOKOYA
LOMA
LOMBI
LOMOTWA
LOMWE
LONDO
LONGARIM
LONGTO
LOPA
LOSAKANYI
LOTSU-PIRI
LOVALE
LOVEDU
LOZI
LUANO
LUBA
LUBA EASTERN
LUBA KASAYI
LUBA SHANKADI
LUBA UPEMBA
LUBA, LUNDA
LUBA, NDEMBU
LUBA, NDEMBU, LUNDA
LUBILA
LUCHAZI
LUGBARE
LUGURU
LULA
LULUBA
LULUWA
LUMBU
LUNDA

LUNDA, CHOKWE
LUNDA, KETE
LUNDA, NDEMBU
LUNGU
LUNTU
LURI
LUSHANGI
LUUNDA
LUYIA
LWALU
LWENA
LWER
LWIMBI
LYANGALILE
LYELE
MA
MAAKA
MAASAI
MAAZA
MABA
MABENDI
MABEZA
MABIHA
MABINZA
MACHINGA
MADA
MADI
MAGHARBA
MAGUZAWA
MAHAFALY
MAHONGWE
MAK
MAKAA
MAKERE
MAKOMA
MAKONDE
MAKUA
MAKWA
MALINKE
MALWAL
MAMBAI
MAMBILA
MAMBWE
MAMPRUSI
MAMVU
MANALA
MANDA
MANDARI
MANDINKA
MANDYAK
MANGAS
MANGAYAT
MANGBELE
MANGBETU
MANGBUTU
MANINKA
MANKANYA
MANO
MANYANGA
MANYIKA
MANZA
MAO SOUTH
MAPOPOI
MARARIT
MARBA
MARFA
MARGHI
MARGHI SOUTH
MARKA
MASAKIN
MASALIT
MASANA
MASHASHA
MASHI
MASMAJE
MASONGO
MASSA
MASSALAT
MATENGO
MATUMBI
MAU
MAURI
MAYOGO
MBA
MBAAMBA
MBAATI
MBAGANI
MBAI BEDIONDO
MBALA
MBANGWE
MBANZA
MBATA

MBEKO
MBELIME
MBELO
MBEMBE
MBEMBRE
MBENE
MBERE
MBESA
MBINSA
MBO
M'BOKO
MBOLE
MBOMOTABA
MBONGO
MBOSHI
MBUGWE
MBUKUSHU
MBULI
MBULU
MBUM
MBUNDA
MBUNGA
MBUUN
MBWELA
MBWELA KOLWE
MBWERA
MDUNDULU
MEDJE
MEDOGO
MENDE
MENING
MERINA
MERU
MESME
MFINU
MFUNU
MIDOB
MIGAAMA
MILTU
MIMI
MINIANKA
MINUNGO
MINUNGU
MITSOGHO
MITTU
MITUKU
MIYA
MOBA
MOBANGO
MOBENGE
MOKOLE
MOLO
MONDOMBE
MONDZOMBO
MONGO
MONO
MONTOL
MOROCCAN
MORU
MOSSI
MOTEMBO
MOYE
MPAMA
MPANGU
MPE
MPEZENI'S NGUNI
MPONGMPONG
MPOTO
MPUT
MPUTU
MPYEMO
MSER
MUBI
MUCHIKONGO
MUKULU
MUMUYE
MUN
MUNDANG
MUNDU
MUNGA
MURLE
MUSGU
MUSSEY
MUSSUMBE
MVELE
MWAGHAVUL
MWENYI
MYANG
MYENE
NABAN
NAFANA
NAGUMI

NALU
NAMBA
NAMJI
NANCERE
NANDE
NANDI
NANDU-TARI
NANDYA
NATENI
NATIORO
NAWDM
NBWERA
NDAAKA
NDALI
NDAM
NDAMBA
NDAU
NDEBELE
NDEMBU
NDEMLI
NDENDEULE
NDENGEREKO
NDENGESE
NDIBU
NDO
NDOGO
NDOLO
NDOOLA
NDULU
NDUNGA
NDZABI
NDZUNDZA
NEFUSA
NEYO
NGABA
NGALA
NGALANGI
NGAM
NGAM GIR BOR
NGAMBAI
NGAMO
NGANDA
NGANDO
NGANDU
NGANDYERA
NGANGELA
N'GARE
NGASA SHAKA
NGATA
NGBAGA
NGBAKA
NGBANDI
NGEENDE
NGENGE
NGENGELE
NGENZA
NGGWAHYI
NGHWELE
NGINDO
NGIZIM
NGOLA
NGOMBE
NGONGI
NGONGO
NGOUNGWONI
NGUL
NGULU
NGUMBA
NGUMBO
NGUNDI
NGUNGULU
NGUNI
NGURIMI
NGWABA
NHAM
NIABWA
NIELLIM
NIMBARI
NINZAM
NJAJGULGULE
NJAMUS
NKANGALA
NKANSI
NKANU
NKHUMBI
NKOLE
NKOMI
NKOYA
NKUKOLI
NKUTSHU
NKWE
NOBIIN

NOLE
NOWOLO
NSAMBA
NSAPO
NSENGA
NSONGO
NTANDU
NTCHAM
NTOMBA
NUBA
NUER
NUMANA
NUNA
NUNGU
NUNU
NUNUMA
NUPE
NWENSHI
NWERA
NYAKYUSA
NYALA
NYALI
NYAMWANGA
NYAMWEZI
NYANEKA-HUMBE
NYANGA
NYANGATOM
NYANGBARA
NYANJA
NYARAFOLO
NYATURU
NYEMBA
NYENGO
NYIHA
NYILAMBA
NYIMANG
NYINDU
NYONG
NYORO
NYULI
NZAKARA
NZANYI
NZIMA
OBOLO
OBULKOM
ODUT
OKAK
OKO-ENI-OSAYEN
OKPE
OKPE-AKUKU
OKPELA
OMBO
OMETO
OMONO
OOLI
OPA
OPAMERI
OPUUO
ORANA
ORING
OROMO
ORON
OTANG
OTUHU
OUASSA
PAI
PALLAKA
PAMBIA
PANA
PANDE
PANI
PAPEL
PARE
PARE, SAA
PATU
PAYE
PENDE
PERE
PERO
PEVE
PHOKA
PIMBWE
PINDA
PINDI
PITI
PIYA
PODJULU
PODZO
POGORO
POKOMO
POMBO
POMO
PONGWE
POTO
PUGULI
PUKU
PUNU
PYAANG
QUILENGUE
RASHAD
REGEIBAT
RENDILL
RIF
RIYAH
RONGA
RUARHA
RUBASA
RUFIJI
RUNGA
RUNYANKOLE
RURI
RUSHA
RUUND
RUWENG
SAAB
SAADI
SABA
SAFWA
SAGALA
SAGARA
SAHEL
SAHO
SAKA
SAKALAVA
SAKATA
SALA
SALA MPASU
SAMBA
SAMBA LEKO
SAMBO
SAMBU
SAMBURU
SAN
SANGA
SANGO
SANGU
SANUSI
SAPAUT
SAR
SARA
SARA GULA
SARA GULAY
SARO
SARUA
SASARU
SAYA
SEBA
SEEKU
SEKE
SENA
SENGA
SENGELE
SERE
SERER
SESE
SH
SHAGAWU
SHAIKIA
SHALL
SHAMBAA
SHANJO
SHATT
SHEBELLE
SHERBRO
SHI
SHIKI
SHILA
SHILLUK
SHINJI
SHIRAWA
SHLUH
SHONA
SHOO
SHUBI
SHUKRIA
SHUWA
SIDAMO
SIDI
SIHANAKA
SIMAA
SINASHA
SINGA
SINYAR
SIRTICAN
SISAALA
SISYA
SIWA
SIZAKI
SO
SOKORO
SOLA
SOLI
SOLONGO
SOLU
SOMRAI
SOMYEWE
SONGHAI
SONGO
SONGOLA
SONGYE
SONINKE
SONJO
SOONDE
SOSSO
SOTHO
SOUTHERN BANGANTU
SUAFA
SUBA
SUBIYA
SUGA
SUK
SUKU
SUKUMA
SUKWA
SUMA
SUMBWA
SUNDI
SUNGOR
SURI
SURUBU
SUSU
SWAKA
SWAZI
SYEMU
TABWA
TAFIRE
TAGWANA
TAHOU
TAJAKANT
TAJUASO
TALE
TALENSI
TAMA
TAMAZIGHT
TAMBAHUAKA
TAMBAS
TAMBERAMA
TAMBO
TAMEZRET
TAMPOLENSE
TANALA
TANDA
TANGA
TANGALE
TANGBAGO
TANKARA
TAPSHIN
TAROK
TASUMSA
TATOG
TAWANA
TAZARAWA
TCHWABO
TEDA
TEFASI
TEGE
TEITA
TEM
TEMNE
TENDA
TEPETH
TERA
TERE
TESO
TETELA
TEVUNDRU
TIBA
TIBEA
TIE
TIENE
TIGRAY
TIGRINYA
TIKAR
TIKUU
TIO
TITU
TIV
TOBANGA
TOGBO
TOKA
TOMA
TONGA
TONGA-INHAMBANE
TONGWE
TOPOKE
TOPOSA
TORAM
TORO
TOROBE
TOTELA
TOUGOURT
TOUYO
TOW
TRARZA
TRIBOUE
TRIPOLITANIAN
TSAAM
TSHIAKA
TSIENIMBALALA
TSIMIHETY
TSONG
TSONGA
TUAT
TUKEN
TUKULOR
TULA
TULAMA
TUMBUKA
TUMBWE
TUMTUM
TUNDJUR
TUNGU
TUNISIAN
TUPURI
TURA
TURKA
TURKANA
TURUMBU
TUSIAN
TUTSI
TYEMBARA
TYENGA
UBANG
UHAMI-IYAYU
UJOGOMA
UKAAN
UKPE
UKPET
UKWUANI
ULED NAIL
UMON
UNGA
UREGU
URHOBO
UTUGWANG
UZEKWE
VAGALA
VAI
VEKHEE
VENDA
VERE
VEZO
VIDUNDA
VILI
VIN
VINZA
VIRA
VIYE
VUMA
VUTE
WAAMA
WADA
WAJA
WALA
WALLAGA
WAMBU
WANDYA
WARGLA
WARJI
WASA
WASI ALAWA
WE
WENYA
WIILE
WILLE
WINIAMA
WINJI
WOLLO
WOLOF
WOM
WONGO
WOYO
XHOSA
YAA
YAELIMA
YAGOUTE
YAHI
YAKA
YAKA, SUKU
YAKOMA
YALIWASA
YAMAIE
YAMANDUNDU
YAMONGO
YANA
YANZI
YAO
YARSE
YASA
YASAMA
YEKE
YELA
YESKWA
YEYE
YIWOM
YOKO
YOMBE
YORUBA
YOWA
YUKUTARE
YUNGUR
ZAGHAWA
ZANAKI
ZANDE
ZANDE ABANDIA
ZANDE AVUNGARA
ZARAMO
ZARI
ZARMA
ZEEM
ZEKARA
ZELA
ZENAGA
ZIBAN
ZIGUA
ZILMANU
ZIMBA
ZINZU
ZOMBO
ZULA

Table S4: Complete key over HIV-1 subtypes shown in Fig. 2D

HIV-B and HIV-C are indicated by stars.

01_AE	13C	50_A1D	A1A2C	AG	GH
01A1	13U	51_01B	A1A2CD	AGJ	GHK
01A1G	14_BG	52_01B	A1A2D	AGU	GJ
01ADF2	15_01B	53_01B	A1A2G	AH	GK
01B	16_A2D	54_01B	A1A3	AHJU	GKU
01BC	16A1	55_01B	A1A6	AJ	GU
01BG	17_BF	56_cpx	A1B	AKU	H
01C	18_cpx	57_BC	A1BD	AU	HJ
01D	18D	58_01B	A1C	★ B	HU
01F2	18G	59_01B	A1CD	BC	J
01G	19_cpx	61_BC	A1CDGKU	BCF1	JK
01GHJKU	19A1	62_BC	A1CG	BCU	JKU
01U	19B	63_02A	A1D	BD	JU
02_AG	20_BG	64_BC	A1DG	BF	K
02A	21_A2D	65_cpx	A1DHK	BF1	KU
02A1	22_01A1	67_01B	A1DK	BF1G	M
02A1A2	22A1U	68_01B	A1DU	BF2	U
02A1G	22DU	69_01B	A1F1	BG	
02A1U	23_BG	70_BF1	A1F2	BK	
02A3	23A1	71_BF1	A1G	BU	
02A6	24_BG	72_BF1	A1GH	★ C	
02AG	25_cpx	73_BG	A1GHU	CD	
02B	26_A5U	74_01B	A1GJ	CDG	
02BD	26C	76_01B	A1H	CF1	
02BG	27_cpx	78_cpx	A1J	CF1U	
02C	28_BF	79_0107	A1K	CG	
02D	29_BF	82_cpx	A1U	CH	
02F2	30_0206	83_cpx	A2	CHU	
02G	31_BC	85_BC	A2B	CJ	
02GK	32_06A6	86_BC	A2C	CJU	
02H	32A6	87_cpx	A2CD	CU	
02HU	32G	90_BF1	A2D	D	
02U	33_01B	102	A2F	DF	
03_AB	34_01B	103	A2G	DF1	
04_cpx	35_AD	107	A2U	DF1G	
05_DF	36_cpx	108	A3	DF2	
06_cpx	37_cpx	113	A3G	DG	
06A1	38_BF	206	A4	DGH	
06G	38_BF1	209	A6	DK	
06U	39_BF	211	A6B	DU	
07_BC	40_BF	218	AB	F	
07B	41_CD	222	ABD	F1	
08_BC	42_BF	609	ABDU	F1F2	
09_cpx	43_02G	708	AC	F1G	
09A1	44_BF	0102A	ACD	F1U	
09A1D	45_cpx	0102A1	ACDJ	F2	
10_CD	45BF1	0122F	AD	F2G	
11_cpx	45F1	1113	ADG	F2K	
11A1	45U	1819	ADU	F2KU	
11AG	46_BF	-	AF	FK	
11C	47_BF	A	AF1	FKU	
12_BF	48_01B	A1	AF2	FU	
13_cpx	49_cpx	A1A2	AF2G	G	

Table S5: Complete key over African languages shown in Fig. 2E

Bantu languages are indicated by stars.

	Adamawa-Ubangian
	Adamawa-Ubangian / Chari-Nile
	Bantoid
★	Bantu
★	Bantu / Bantu
	Berber
	Chadic
	Chadic / Cushitic
	Chadic / Fufulde
	Chari-Nile
	Chari-Nile / Nilotic
	Cushitic
	Fufulde
	Fufulde / Adamawa-Ubangia
	Fur
	Gbaya
	Khoi: Nama, Bergdama
	Kordofanian
	Kru
	Kwa
	Maban
	Malagasy
	Miscellaneous / Unclassified
	Nilotic
	Nilotic / Bantoid
	Nilotic / Bantu
	Northern Mande
	Other
	Saharan
	Saharan / Cushitic
	Saharan / Nilotic
	San
	Sandawe
	Semitic: Arab, Bedouin
	Songhai
	Southern Mande
	Voltaic
	West Atlantic

Table S6: HLA class I allele frequencies in worldwide populations

	ZA Blacks	ZA Zulu	US African	US Caribbean	Thailand	US Chinese	US Caucasian	UK central
**A*02:01**	0.083	0	0.123	0.111	0.255	0.0946	0.2755	0.2697
**B*07:02**	0.046	0.07	0.073	0.073	0.038	0.0079	0.1306	0.1391
**C*07:02**	0.058	0.08	0.073	0.067	0	0.1945	0.1415	0.155
**A*30:01**	0.101	0.1	0.068	0.072	0.013	0.0274	0.013	0.011
**B*42:01**	0.089	0.11	0.053	0.053	0	0.0001	0.0003	0.0019
**C*17:01**	0.111	0.05	0.068	0.068	0	0.0005	0.0088	0.004
**B*58:02**	0.094	0.11	0.042	0.032		0	0.0001	0
**C*06:02**	0.149	0.15	0.087	0.072	0	0.0447	0.0932	0.11
**Population**	ZA Blacks	ZA Zulu	US African	US Caribbean	Thailand	US Chinese	US Caucasian	UK central
**Sum of HLA variants common in Caucasian**	0.187	0.15	0.269	0.251	0.293	0.297	0.5476	0.5638
**Sum of HLA variants common in Africans**	0.544	0.52	0.318	0.297	0.013	0.0727	0.1154	0.1269

The individual HLA alleles frequencies of HLA variants that contributed most to the first two principal components (PCs) were also found in common African and Caucasian haplotypes. Here we show the individual frequencies of each HLA variant and the sum of the variants that can form haplotypes in Africans and Caucasians. The data derive from (Gonzalez-Galarza et al. 2015), and the South Africa (ZA) Zulu data derive from the Females Rising through Education, Support, and Health (FRESH) project.

Table S7: Overview of the amino acid variation at SSP in HIV-1 subtype consensus sequences

HIV subtype consensus	Position 27 amino acid	Position 41	Position 116	Position 120	Position 128
**HIV-A1**	I	S	G	G	D
**HIV-A2**	V	T	G	S	E
**HIV-B**	V	S	G	N	E
**HIV-C**	I	T	A	S	D
**HIV-D**	I	S	G	S	E
**HIV-F1***	I	S	Q	S	D
**HIV-G**	V	S	R	S	E
**HIV-H***	V	S	A	G	D
**HIV-K***	I	S	T	S	E

(From www.hiv.lanl.gov/content/sequence/NEWALIGN/align.html, (Foley et al. 2018))

*HIV-F1 predominates in South America, especially in Brazil, Uruguay, and Argentina, HIV-H is found in Central Africa, Eastern Europe, and Central Asia, and HIV-K is found in Central Africa and Pakistan (Khan et al. 2018) at low frequency.

The subtype distribution of HIV-F1, HIV-H and HIV-K outside of Africa – and the presence of, e.g., HIV-C in India and China - is due to founder effects. The evolution of these subtypes over time in non-African countries is unknown due to limited sampling and the brevity of the ongoing HIV epidemic (Buonaguro et al. 2007; Hemelaar et al. 2011; Osmanov et al. 2002). Overall, HIV-F (0.59%), HIV-H (0.17%), HIV-J (0.14%), and HIV-K (0.04%) together cause fewer than 1% of HIV-1 infections worldwide (Buonaguro et al. 2007; Hemelaar et al. 2011). No consensus sequences for HIV-J p24Gag are available from the Los Alamos HIV sequence database (Foley et al. 2018).

Table S8: LANL HIV database patient IDs of patients with imputed four digit HLAs

B*27 → B*27:05:

402, 3393, 3394, 10542, 11222, 13225, 15792, 19252, 19542, 19878, 22972, 22973, 22974, 22975, 22982, 22984, 22986, 23077, 27220, 34369, 36112, 36113, 49996

B*35 → B*35:01:

24029, 30977, 35886, 36113, 49994, 51971

B*57 → B*57:01:

10487, 10488, 10489, 10490, 10491, 10492, 10493, 10494, 10495, 10496, 10497, 10498, 10499, 10500, 10501, 10502, 10503, 10504, 10505, 10506, 10507, 10508, 10509, 10510, 11222, 13224, 15396, 15397, 19539

References

Abi-Rached, L., et al. (2011), 'The shaping of modern human immune systems by multiregional admixture with archaic humans', *Science,* 334 (6052), 89-94.

Beloukas, A., et al. (2016), 'Molecular epidemiology of HIV-1 infection in Europe: An overview', *Infect Genet Evol,* 46, 180-89.

Buonaguro, L., Tornesello, M. L., and Buonaguro, F. M. (2007), 'Human immunodeficiency virus type 1 subtype distribution in the worldwide epidemic: pathogenetic and therapeutic implications', *J Virol,* 81 (19), 10209-19.

Diez-Fuertes, F., Cabello, M., and Thomson, M. M. (2015), 'Bayesian phylogeographic analyses clarify the origin of the HIV-1 subtype A variant circulating in former Soviet Union's countries', *Infect Genet Evol,* 33, 197-205.

Draenert, R., et al. (2004), 'Immune selection for altered antigen processing leads to cytotoxic T lymphocyte escape in chronic HIV-1 infection', *J Exp Med,* 199 (7), 905-15.

Faroudi, M., et al. (2003), 'Lytic versus stimulatory synapse in cytotoxic T lymphocyte/target cell interaction: manifestation of a dual activation threshold', *Proc Natl Acad Sci U S A,* 100 (24), 14145-50.

Foley, Brian, et al. (2018), *HIV Sequence Compendium 2018* (Theoretical Biology and Biophysics, Los Alamos National Laboratory, Los Alamos, New Mexico 87545 U.S.A.LA-UR-18-25673).

Gonzalez-Galarza, F. F., et al. (2015), 'Allele frequency net 2015 update: new features for HLA epitopes, KIR and disease and HLA adverse drug reaction associations', *Nucleic Acids Res,* 43 (Database issue), D784-8.

Goulder, P. J. and Watkins, D. I. (2008), 'Impact of MHC class I diversity on immune control of immunodeficiency virus replication', *Nat Rev Immunol,* 8 (8), 619-30.

Goulder, P. J. and Walker, B. D. (2012), 'HIV and HLA class I: an evolving relationship', *Immunity,* 37 (3), 426-40.

Gragert, L., et al. (2013), 'Six-locus high resolution HLA haplotype frequencies derived from mixed-resolution DNA typing for the entire US donor registry', *Hum Immunol,* 74 (10), 1313-20.

Hall, H. I., et al. (2008), 'Estimation of HIV incidence in the United States', *JAMA,* 300 (5), 520-9.

Hemelaar, J., et al. (2011), 'Global trends in molecular epidemiology of HIV-1 during 2000-2007', *AIDS,* 25 (5), 679-89.

Khan, S., et al. (2018), 'HIV-1 genetic diversity, geographical linkages and antiretroviral drug resistance among individuals from Pakistan', *Arch Virol,* 163 (1), 33-40.

Kijak, G. H., et al. (2009), 'HLA class I allele and haplotype diversity in Ugandans supports the presence of a major east African genetic cluster', *Tissue Antigens,* 73 (3), 262-9.

Lucchiari-Hartz, M., et al. (2003), 'Differential proteasomal processing of hydrophobic and hydrophilic protein regions: contribution to cytotoxic T lymphocyte epitope clustering in HIV-1-Nef', *Proc Natl Acad Sci U S A,* 100 (13), 7755-60.

Osmanov, S., et al. (2002), 'Estimated global distribution and regional spread of HIV-1 genetic subtypes in the year 2000', *J Acquir Immune Defic Syndr,* 29 (2), 184-90.

Paximadis, M., et al. (2012), 'Human leukocyte antigen class I (A, B, C) and II (DRB1) diversity in the black and Caucasian South African population', *Hum Immunol,* 73 (1), 80-92.

Prejean, J., et al. (2011), 'Estimated HIV incidence in the United States, 2006-2009', *PLoS One,* 6 (8), e17502.

Tenzer, S., et al. (2014), 'HIV-1 adaptation to antigen processing results in population-level immune evasion and affects subtype diversification', *Cell Rep,* 7 (2), 448-63.
Tenzer, S., et al. (2009), 'Antigen processing influences HIV-specific cytotoxic T lymphocyte immunodominance', *Nat Immunol,* 10 (6), 636-46.
